# Association between hemoglobin glycation index and cognitive function: Evidence in the elderly

**DOI:** 10.1371/journal.pone.0338613

**Published:** 2026-05-15

**Authors:** Liang Ge, Yapeng Guo, Xiangjun Xu, Junfeng Xu, Menghan Hu

**Affiliations:** Department of Neurology, Yijishan Hospital of Wannan Medical College, Wuhu, China; Sichuan University, CHINA

## Abstract

**Background:**

Cognitive impairment has been a major public health challenge. This study aims to estimate the association of hemoglobin glycation index (HGI) with cognitive function in U.S. older adults.

**Methods:**

In this cross-sectional study, data from the National Health and Nutrition Examination Survey (NHANES, 2011–2014) was obtained. Cognitive function was assessed based on the three scales [Digit Symbol Substitution Test (DSST), Animal Fluency Test (AFT), and Consortium to Establish a Registry for Alzheimer’s disease (CERAD)], along with a composite Z-score derived from the sum of the Z-scores of these three assessments. We used weighted univariate and multivariate linear regression to estimate the unstandardized β coefficient and 95% confidence interval (CI) of the association between HGI levels and cognitive function.

**Results:**

Our study comprised a total of 1,406 subjects. As compared with the second tertile of HGI, the lowest tertile had a lower Z-score [β = −0.13 (95% CI: −0.25, −0.01)] and CERAD score [β = −1.07 (95% CI: −1.93, −0.21)] in the fully adjusted model. For the highest tertiles of HGI (*vs.* second tertile), Z-score and DSST decreased by 0.16 (95%CI: −0.31, −0.01) and 3.09 (95%CI: −6.00, −0.18), respectively. Females in the lowest HGI tertile (*vs.* second HGI tertile) exhibited a decline in cognitive function scores (Z-score, CERAD, AFT; all *P* < 0.05), while males in the highest tertile of HGI (*vs.* second HGI tertile) showed decreased cognitive function scores (Z-score, DSST; all *P* < 0.05). In addition, Z-score and CERAD scores also decreased (all *P* < 0.05) in the highest HGI tertile (*vs.* second HGI tertile) among non-users of antidiabetic drugs.

**Conclusion:**

Both lower and higher HGI levels are associated with cognitive decline. Lower HGI is related to poorer learning and memory, while higher HGI is associated with executive function impairment. Future longitudinal studies are need to verify whether changes in HGI levels can be used as an early warning indicator for cognitive decline.

## 1. Introduction

Cognitive impairment can be defined as a significant decline or loss in an individual’s cognitive functions, encompassing memory, language, attention, executive function, and visuospatial processing [1]. Deterioration in cognitive abilities is seen as the precursor of Alzheimer’s disease [2] and other dementias [3], which impose a notable effect on the overall well-being, autonomy, and health of senior individuals [4]. Alzheimer’s disease impacts approximately 6.9 million U.S. adults aged ≥ 65 and older, making it the fifth leading cause of death [5]. How to effectively identify and manage age-related cognitive decline has become an urgent public health problem to be solved.

As common metabolic abnormalities in the elderly, impaired glucose metabolism, and dysfunctional insulin activity are involved in the underlying mechanisms of cognitive decline associated with type 2 diabetes mellitus (DM) [6]. Maan et al. [7] found that high glycated hemoglobin (HbA1c)/uncontrolled DM and DM duration resulted in cognitive impairment. Although fasting plasma glucose (FPG) and HbA1c are commonly used in the diagnosis and management of DM in clinical practice, evidence suggests that the HbA1c level is not completely consistent with the FPG level [8,9]. Factors such as the mean lifespan of red blood cells, glucose transmembrane gradients across cell membranes, and enzyme abnormalities may influence HbA1c levels [10,11], indicating HbA1c measurements may not fully reflect glycemic metabolic status. Hemoglobin glycation index (HGI) has been introduced to measure the extent and direction of the variance between actual and expected HbA1c levels in individuals, aiming to characterize individual susceptibility to hemoglobin glycation [8]. Elevated HGI is associated with metabolic abnormalities, inflammation, and oxidative stress [12,13], while a low HGI may result from elevated FPG and low HbA1c due to stress-induced hyperglycemia [14,15]. Previous studies showed a U-shaped relationship of HGI levels with adverse cardiovascular events in patients with diabetes and coronary heart disease [15–17], and an L-shaped association of HGI levels with 30-day and in-hospital mortality in patients with ischemic stroke [18]. Recent findings have shown an association between increased HGI with shorter telomere length [14], which in turn is connected to cognitive decline [19]. However, the relationship of HGI with cognitive function remains unclear.

Our study aims to provide some evidences of the association between HGI levels and cognitive function in older adults. We believe that our findings can lead to better ways of identification and monitoring of cognitive impairment risk in the elderly through the use of readily accessible biomarkers.

## 2. Methods

### 2.1. Study population and design

As a representative, long-term program, the National Health and Nutrition Examination Survey (NHANES) database was designed to evaluate the health and nutritional conditions of U.S. citizens. The health data of participants were obtained through home visits and physical examinations. Data acquisition and details, as well as the design of the NHANES program, can be accessed at https://www.cdc.gov/nchs/nhanes/index.htm. The project was approved by the Ethics Review Board of National Center for Health Statistics (Protocol #2011−17, available at https://www.cdc.gov/nchs/nhanes/irba98.htm). Each survey participant signed a consent form.

**Fig 1:**
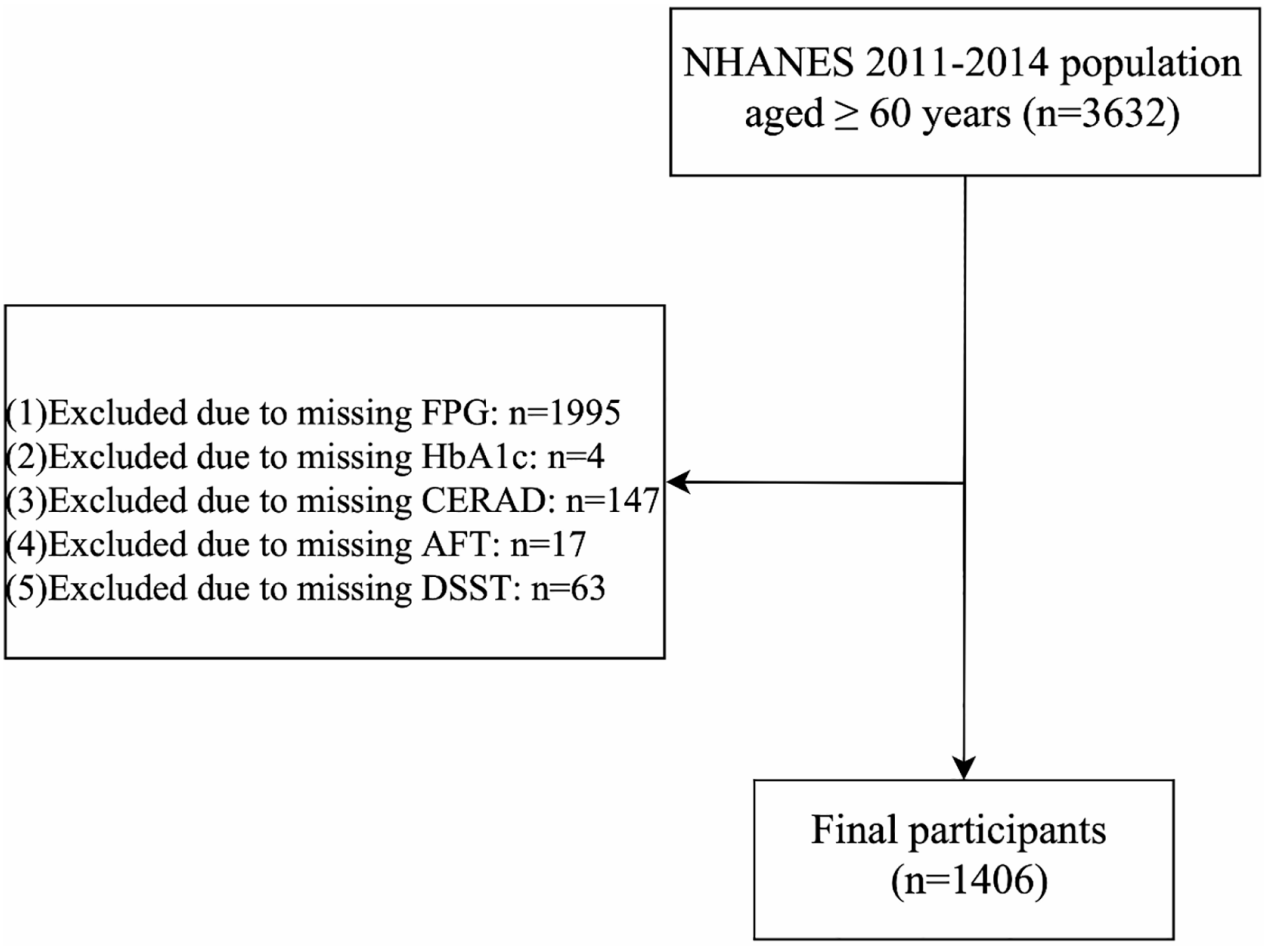
Flow chart of the participants’ selection.

Participants with cognitive function tests were included from two NHANES cycles (2011–2012 and 2013–2014). Initially, 3,632 participants ≥ aged 60 years were recruited, excluding missing data on FPG (n = 1,995), HbA1c (n = 4), the consortium to establish a registry for Alzheimer’s disease (CERAD) (n = 147), the animal fluency test (AFT) (n = 17) and the digit symbol substitution test (DSST) (n = 63), 1,406 participants were finally enrolled. The flowchart of participant selection is shown in Fig 1. The sensitivity analysis comparing baseline characteristics between included and excluded cohorts was presented in S1 Table in S1 File.

### 2.2. Definition of HGI

**Fig 2:**
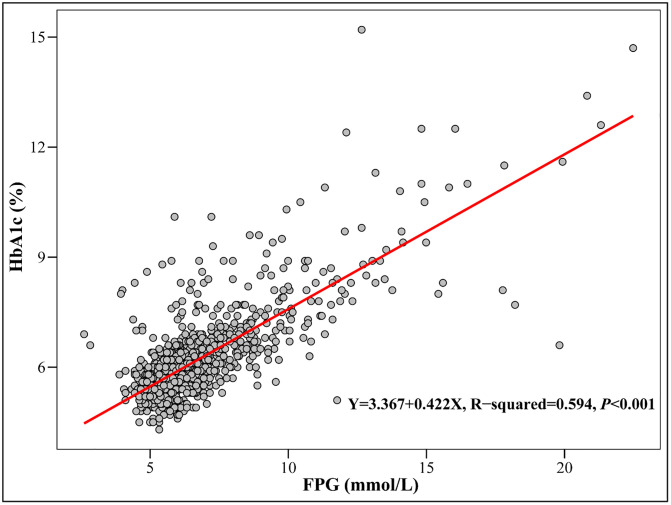
Correlation of FPG with HbA1c.

The HGI calculated relied on the availability of HbA1c and FPG data from participants. Blood samples of participants were obtained in the morning after fasting (Fasting for 8–24 hours before blood sampling). Based on the participants enrolled in this study, an equation between FPG and HbA1c was constructed [predicted HbA1c (%) = 0.422 × FPG (mmol/L) + 3.367, R^2^ = 0.594] (Fig 2). HGI was calculated by the following equation [20]: HGI = measured HbA1c - predicted HbA1c. All participants were grouped by tertiles of HGI: Tertile 1 (HGI < −0.226), Tertile 2 (−0.226 ≤ HGI < 0.119), Tertile 3 (≥ 0.119).

### 2.3. Cognitive Function

In NHANES 2011–2014, cognitive function tests were performed among adults aged ≥ 60 years. The CERAD assesses participants’ immediate and delayed learning abilities for new verbal information through three sequential learning trials and a delayed recall. In each trial, participants were tasked with reading aloud 10 unrelated words and were required to recall them immediately after the presentation or 10 minutes later. To evaluate verbal fluency and production, the AFT required participants to list as many animals as possible starting with a designated letter within a time limit of one minute. In DSST, participants were required to match different symbols to the corresponding numbers in a limited time to assess information processing speed and task execution ability. Finally, the composite-Z score was calculated from the sum of the Z scores [(individual test score – mean score)/standard deviation] of these three tests (DSST, AFT, and CERAD). For each cognitive function test, higher scores represented better cognitive function.

### 2.4. Covariates

Potential covariates included age (continuous, years), sex (male, female), race (non-Hispanic White, non-Hispanic Black, Mexican American, other race), poverty income ratio (PIR; < 1, ≥ 1), marriage (married or living with partner, living alone), education level (high school and below, above high school), physical activity [metabolic equivalent of task (MET)/ minutes per week; < 450, 450–750, ≥ 750, unknown], smoking (never, former, current), hypertension (no, yes), antidiabetic drug used (no, yes), dyslipidemia (no, yes), heavy drinking (no, yes, unknown), body mass index (BMI; kg/m^2^; < 25, ≥ 25), depression (no, yes), chronic kidney disease (CKD; no, yes), stroke (no, yes), antiparkinson agents (no, yes), anxiolytics sedatives hypnotics (no, yes), psychotherapeutic agents (no, yes), total energy (continuous, kcal), carbohydrate (continuous, gm), protein (continuous, gm), total fat (continuous, gm), total sugars (continuous, gm). Please refer to our S1 File for detailed variable definitions.

### 2.5. Statistical analysis

To address the multistage design, we employed SURVEY procedures which the National Institutes of Health (NCHS) recommended. The procedures incorporate the morning fasting subsample 2-year weights (WTSAF2YR), strata (SDMVSTRA), along primary sampling units (SDMVPSU) into the weighted analyses. The linearity, normality and homoscedasticity of residuals, and residual plots were shown in S1–S4 Fig. All figures (S1A–S4A Figs) showed that the red line was roughly a straight line parallel to the X-axis. That is, the residuals were not affected by the independent variables. In Figs (S1B–S4B), the residuals were normally distributed. The red line was roughly a straight line parallel to the X-axis, indicating that the variance of the residuals was homogeneous (S1C–S4C Figs). No outliers were found in residual plots (S1D–S4D Figs). Mean ± standard error and the weighted one-way analysis of variance (ANOVA) were used in the descriptive analysis of continuous variables. Frequencies (percentage) [n (%)] and the Rao-Scott Chi-square test were used for the categorical variables. The missing data was evaluated by Little’s MCAR test. The result of *P*-value was 0.157, indicating the missing data conformed to complete random missing. Missing data were handled using the multiple imputation method based on chained equations under the missing at random. Five imputed datasets were generated and analyzed separately, and the results were pooled according to Rubin’s rules. The missing values were presented in Table S2 in S1 File. The sensitivity analysis on comparison of variables before and after data interpolation was shown in Table S3 in S1 File.

All results were analyzed by R software (version 4.3.3), with a significant set at a *P*-value < 0.05. The restricted cubic spline (RCS) curves were used to assess the nonlinear correlations of HGI levels and cognitive function scores. The initial weighted univariate linear regression model incorporated all potential variables and those with a *P* < 0.05 were then entered into a backward stepwise regression analysis to identify the relevant covariates (Table S4-S7 in S1 File). The AIC values of Z-score, CERAD, AFT, and DSST were 3050.86, 8760.71, 8437.54, and 11134.10, respectively. The multicollinearity assessment for covariates were presented in Table S8 in S1 File. The results show that there is no collinearity among covariates. Subsequently, the associations of cognitive function (CERAD, AFT, DSST, and cognitive Z-score) with HGI were evaluated by the univariate and multivariable linear regression models. The unstandardized β and confidence interval (CI) was calculated. An in-depth exploration of the association between HGI and cognitive function was conducted using subgroup analysis (grouped participants according to sex and the use of antidiabetic drugs). The interaction terms of subgroups were further tested. Due to multiple testing may increase the risk of Type I error, a Bonferroni correction was considered to assess the results of all analyses. The Bonferroni correction has a significance level of α’ = 0.05/n, where n is the number of subgroups to be compared (n = 4 in the main analyses, and n = 16 in the subgroups), resulting in an adjusted significance level of α’ = 0.0125 or 0.003125. We considered the result in the subgroups still statistically significant only if the *P*-value <0.003125.

## 3. Results

### 3.1. Characteristics of study participants

This cross-sectional study enrolled 1,406 samples. The characteristics of the included participants were presented in Table 1. The HGI was categorized into Tertile 1 (HGI < −0.266), Tertile 2(−0.266 ≤ HGI < 0.119), and Tertile 3 (HGI ≥ 0.119). The mean (±SD) age of the participants was 69.09 (±0.26) years with most participants being female (n = 715, 55.74%) and non-Hispanic White (n = 708, 79.26%). Our results exhibited differences in Z-scores, CERAD scores, AFT scores, and DSST scores among different HGI level groups were observed (all *P* < 0.05). Among the different HGI level groups, differences in nutrient intake (total energy, carbohydrates, protein, total fat, and total sugars), as well as glycation indices (HbA1c and FPG), were also found (all *P* < 0.05). The race, PIR, education level, PA, antidiabetic drug use, heavy drinking, BMI, and CKD history were statistical differences among the tree groups (all *P* < 0.05).

**Table 1:** Characteristics of the study population by HGI level tertiles.

Variables	Total (n = 1406)	Tertile 1 (N = 466)	Tertile 2 (n = 470)	Tertile 3 (n = 470)	*P*
Age (years)	69.09 ± 0.26	68.89 ± 0.34	69.05 ± 0.40	69.42 ± 0.38	0.629
Sex, n (%)					0.001
Male	691 (44.26)	265 (53.99)	208 (37.75)	218 (40.56)	
Female	715 (55.74)	201 (46.01)	262 (62.25)	252 (59.44)	
Race, n (%)					<0.001
Non-Hispanic White	708 (79.26)	260 (83.94)	265 (83.42)	183 (67.92)	
Non-Hispanic Black	289 (8.49)	80 (6.07)	75 (6.50)	134 (14.16)	
Mexican American	124 (3.46)	41 (3.48)	39 (2.67)	44 (4.48)	
Others	285 (8.79)	85 (6.52)	91 (7.42)	109 (13.44)	
PIR, n (%)					0.002
< 1	226 (8.69)	58 (6.75)	67 (6.99)	101 (13.35)	
≥ 1	1180 (91.31)	408 (93.25)	403 (93.01)	369 (86.65)	
Marriage, n (%)					0.973
Married or living with partner	874 (68.48)	293 (69.00)	294 (68.27)	287 (68.10)	
Living alone	532 (31.52)	173 (31.00)	176 (31.73)	183 (31.90)	
Education, n (%)					0.004
High school and below	702 (39.53)	205 (33.88)	222 (37.56)	275 (49.21)	
Above high school	704 (60.47)	261 (66.12)	248 (62.44)	195 (50.79)	
PA, (MET/ minutes per week), n (%)					0.027
< 450	158 (9.94)	46 (9.42)	61 (11.77)	51 (8.19)	
450-750	136 (9.24)	36 (6.84)	43 (8.50)	57 (13.22)	
≥ 750	630 (48.07)	223 (51.34)	217 (50.51)	190 (40.76)	
Unknown	482 (32.76)	161 (32.41)	149 (29.22)	172 (37.83)	
Smoking, n (%)					0.739
Never	699 (49.01)	223 (46.59)	249 (51.75)	227 (48.48)	
Former	539 (40.52)	192 (43.30)	164 (38.01)	183 (40.32)	
Current	168 (10.47)	51 (10.11)	57 (10.24)	60 (11.20)	
Hypertension, n (%)					0.103
No	256 (20.80)	89 (19.62)	99 (24.62)	68 (17.27)	
Yes	1150 (79.20)	377 (80.38)	371 (75.38)	402 (82.73)	
Antidiabetic drug, n (%)					<0.001
No	1091 (80.44)	384 (85.15)	416 (89.85)	291 (62.20)	
Yes	315 (19.56)	82 (14.85)	54 (10.15)	179 (37.80)	
Dyslipidemia, n (%)					0.110
No	209 (15.03)	86 (18.61)	69 (12.81)	54 (13.43)	
Yes	1197 (84.97)	380 (81.39)	401 (87.19)	416 (86.57)	
Heavy drinking, n (%)					0.002
No	917 (67.01)	293 (61.83)	309 (67.86)	315 (72.40)	
Yes	218 (17.22)	86 (23.48)	77 (17.62)	55 (8.83)	
Unknown	271 (15.77)	87 (14.69)	84 (14.52)	100 (18.77)	
BMI, (kg/m^2^), n (%)					0.021
< 25	388 (27.51)	121 (23.17)	152 (35.08)	115 (23.04)	
≥ 25	1018 (72.49)	345 (76.83)	318 (64.92)	355 (76.96)	
Depression, n (%)					0.370
No	1273 (91.95)	423 (91.60)	429 (93.58)	421 (90.26)	
Yes	133 (8.05)	43 (8.40)	41 (6.42)	49 (9.74)	
CKD, n (%)					0.014
No	1008 (75.66)	339 (77.35)	357 (80.32)	312 (67.44)	
Yes	398 (24.34)	127 (22.65)	113 (19.68)	158 (32.56)	
Stroke, n (%)					0.253
No	1305 (93.31)	428 (93.62)	444 (94.45)	433 (91.43)	
Yes	101 (6.69)	38 (6.38)	26 (5.55)	37 (8.57)	
Antiparkinson agents, n (%)					0.212
No	1384 (98.45)	456 (97.66)	465 (99.30)	463 (98.34)	
Yes	22 (1.55)	10 (2.34)	5 (0.70)	7 (1.66)	
Anxiolytics sedatives hypnotics, n (%)					0.556
No	1319 (93.85)	432 (93.12)	441 (94.74)	446 (93.62)	
Yes	87 (6.15)	34 (6.88)	29 (5.26)	24 (6.38)	
Psychotherapeutic agents, n (%)					0.911
No	1176 (81.08)	383 (80.54)	391 (81.63)	402 (81.04)	
Yes	230 (18.92)	83 (19.46)	79 (18.37)	68 (18.96)	
Total energy (kcal)	1887.92 ± 30.89	2009.99 ± 47.75	1903.64 ± 48.76	1713.96 ± 38.92	<0.001
Carbohydrate (gm)	222.63 ± 4.00)	234.58 ± 6.44	222.77 ± 5.92	207.43 ± 6.09	0.012
Protein (gm)	74.16 ± 1.53	76.13 ± 1.53	76.04 ± 2.62	69.22 ± 2.16	0.022
Total fat (gm)	75.19 ± 1.50	81.08 ± 2.66	76.16 ± 2.68	66.53 ± 1.92	<0.001
Total sugars (gm)	94.66 ± 1.99	100.15 ± 3.96	95.16 ± 2.96	87.10 ± 3.33	0.040
Z-score	0.38 ± 0.05	0.40 ± 0.07	0.56 ± 0.06	0.13 ± 0.07	<0.001
CERAD-WLT	26.34 ± 0.38	25.95 ± 0.52	27.27 ± 0.36	25.63 ± 0.50	0.001
AFT	18.09 ± 0.22	18.04 ± 0.43	18.70 ± 0.27	17.34 ± 0.43	0.008
DSST	51.92 ± 0.77	52.80 ± 1.02	54.69 ± 1.14	47.17 ± 1.24	<0.001
HbA1c (%)	5.96 ± 0.05	5.57 ± 0.06	5.78 ± 0.03	6.69 ± 0.09	<0.001
FBG (mmol/L)	6.23 ± 0.09	6.46 ± 0.12	5.86 ± 0.07	6.42 ± 0.15	<0.001
HGI (%)	−0.04 ± 0.03	−0.52 ± 0.02	−0.06 ± 0.00	0.61 ± 0.04	<0.001

Mean ± standard error or frequencies (percentage) was used in the data description. HGI, hemoglobin glycation index; PIR, poverty income ratio; PA, physical activity; BMI, body mass index; CKD, chronic kidney disease; CERAD, Consortium to Establish a Registry for Alzheimer’s disease; AFT, Animal Fluency Test; DSST, Digit Symbol Substitution Test; HbA1c, glycated hemoglobin; FBG, fasting plasma glucose. *P*-values were obtained using the weighted one-way analysis of variance or the Rao-Scott Chi-square test, respectively.

### 3.2. Association between HGI and cognitive function

**Fig 3:**
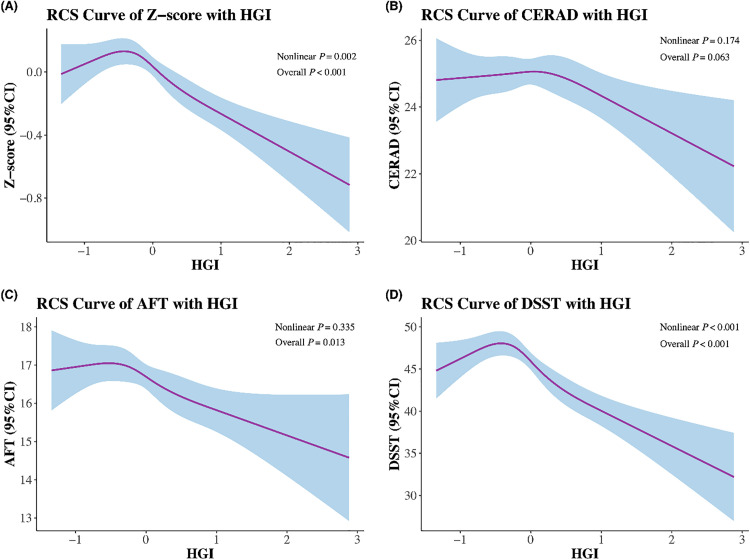
The nonlinear correlation of cognitive function scores with HGI. A. Z-score, B. CERAD, C. AFT, D. DSST.

Tables S3-S6 in S1 File exhibited the process of covariables selection. In adjusted model (Table 2), participants with the lowest tertile of HGI had lower Z-score scores [β = −0.13 (95% CI: −0.25, −0.01)] and CERAD scores [β = −1.07 (95%CI: −1.93, −0.21)] than those in the Tertile 2 group. The Z-score [β = −0.16 (95% CI: −0.31, −0.01)] and DSST (β = −3.09 (95% CI: −6.00, −0.18)] in the highest tertile of the HGI group were decreased than those in the Tertile 2 group. Additionally, when HGI was included as a continuous variable, HGI levels were not associated with Z-score, CERAD, AFT and DSST (all P > 0.05). The nonlinear correlation of cognitive function scores (Z-score, CERAD, AFT and DSST) with HGI was displayed in Fig 3. The nonlinear correlation was found between HGI and Z-score, DSST (P < 0.05).

**Table 2:** The associations between different HGI levels and cognitive function in the older population.

Variable	Z-score		CERAD		AFT		DSST	
	Crude model	Adjusted model	Crude model	Adjusted model	Crude model	Adjusted model	Crude model	Adjusted model
	β (95%CI)	β (95%CI)	β (95%CI)	β (95%CI)	β (95%CI)	β (95%CI)	β (95%CI)	β (95%CI)
HGI								
Tertile 2	Ref	Ref	Ref	Ref	Ref	Ref	Ref	Ref
Tertile 1	−0.16 (−0.32, 0.00)	−0.13 (−0.25, −0.01)^***^	−1.32 (−2.29, −0.35)^**^	−1.07 (−1.93, −0.21)^**^	−0.66 (−1.58, 0.25)	−0.75 (−1.60, 0.10)	−1.89 (−4.59, 0.82)	−1.23 (−3.42, 0.96)
Tertile 3	−0.42 (−0.59, −0.25)^***^	−0.16 (−0.31, −0.01)^*^	−1.64 (−2.48, −0.80)^*^	−0.63 (−1.44, 0.18)	−1.37 (−2.34, −0.39)^**^	−0.35 (−1.18, 0.49)	−7.52 (−10.63, −4.41)^***^	−3.09 (−6.00, −0.18)^**^
HGI (continuous)	−0.21 (−0.36, −0.06)^**^	−0.06 (−0.16, 0.04)	−0.43 (−1.06, 0.20)	0.10 (−0.49, 0.70)	−0.66 (−1.57, 0.25)	−0.02 (−0.58, 0.55)	−4.22 (−6.74, −1.70)^**^	−1.67 (−3.41, 0.07)

HGI, hemoglobin glycation index; CERAD, Consortium to Establish a Registry for Alzheimer’s Disease; AFT, Animal Fluency Test; DSST, Digit Symbol Substitution Test.

*: *P* < 0.05; **: *P* < 0.01; ***: *P* < 0.001.

Crude model adjusted for none.

Adjusted model (Z-score): adjustment for age, sex, race, PIR, education, smoking, chronic kidney disease, psychotherapeutic agents, and total energy.

Adjusted model (CERAD): adjustment for age, sex, race, education, hypertension, chronic kidney disease, anxiolytics sedatives hypnotics.

Adjusted model (AFT): adjustment for age, race, education, physical activity, depression, and antiparkinson agents.

Adjusted model (DSST): adjustment for age, sex, race, PIR, education, smoking, chronic kidney disease, psychotherapeutic agents, and total energy.

### 3.3. Subgroup analysis

**Fig 4:**
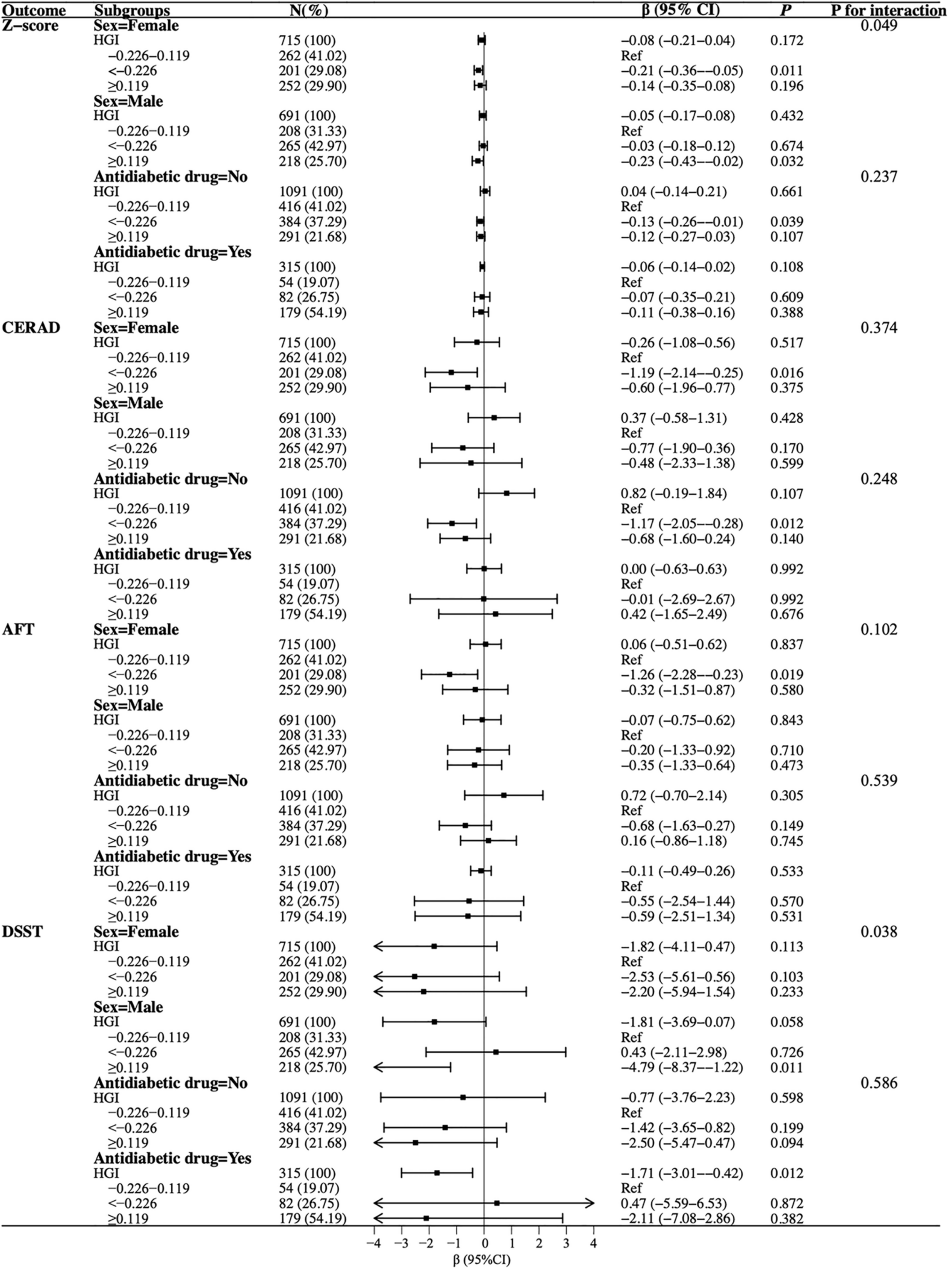
Subgroup analyses for the association of HGI with cognitive function.

Further exploratory analysis of the association between HGI and cognitive function was evaluated by the stratification of sex and antidiabetic drugs used (Fig 4). In the subgroup of females, the lowest tertile (*vs.* Tertile 2) of HGI was associated with decreased scores of Z-scores, CERAD, and AFT by 0.21 (95%CI: −0.36, −0.05), 1.19 (95%CI: −2.14, −0.25), and 1.26 (95%CI: −2.28, −0.23), respectively. However, in male participants, 0.23 (95%CI: −0.43, −0.02) and 4.79 (95%CI: −8.37, −1.22) decreases in Z-score and DSST scores were observed for the highest tertile of HGI levels (*vs.* Tertile 2). Compared with the Tertile 2 of the HGI level, participants who did not use antidiabetic drugs had an increased Z-score [β = −0.13 (95%CI: −0.26, −0.01)] and CERAD scores [β = −1.17 (95%CI: −2.05, −0.28)] in the lowest tertile of the HGI level. For participants using antidiabetic drugs, each increase in HGI was associated with a decrease of 1.71 (95% CI: −3.01, −0.42) in the DSST score.

## 4. Discussion

This representative cross-sectional study of the U.S. elderly population found associations between lower or higher levels of HGI and cognitive function decline (Z-score). Specifically, lower HGI was associated with impaired learning and memory (CERAD), while higher HGI was associated with impaired executive function (DSST). We also observed lower CERAD scores and AFT scores for females or non-users of antidiabetic drug with lower HGI, and lower DSST scores for males with higher HGI.

Glucose serves as the primary energy source for the brain, and changes in blood glucose concentration can influence individual brain function [21]. A meta-analysis indicated a non-linear association between FPG and cognitive impairment, while elevated 2-hour post-load glucose and HbA1c levels were related to an increased developing dementia risk. [22]. A community-based study of 13,351 adults found associations between diabetes, pre-diabetes, poorly controlled diabetes, and a longer duration of diabetes with a lower Z-score [23]. Moreover, the association between hyperglycemic status and cognitive decline appears to be primarily driven by declines in DSST and AFT [23,24]. Approximately 30 million people in the U.S. might experience a clinical mismatch between their HbA1c and glucose levels, potentially resulting in either misdiagnosis or inappropriate management related to HbA1c [25]. Low HbA1c or low FPG does not necessarily indicate good blood glucose control [26], which may not accurately reflect individual glucose variability and long-term stability. Cognitive decline was associated with both lower and higher levels of HGI in this study. Unlike previous studies, we found that lower HGI was associated with impaired learning and memory, whereas higher HGI was associated with impaired executive function, indicating that the effects of different glycemic states on cognition may involve distinct cognitive domains.

Elevated HGI levels were associated with greater production of advanced glycation end-products (AGEs) [27]. Increased AGEs can induce the production of reactive oxygen species (ROS) and activate stress-related signal transduction molecules (p38 mitogen-activated protein kinase and c-Jun N-terminal kinase) [28], resulting in abnormal hyperphosphorylation of tau, which is also a hallmark of Alzheimer’s dementia [29]. A mouse model found that oxidative stress induced by high glucose or AGEs inhibited mitochondrial autophagy in hippocampal neurons through Kelch-like ECH-associated protein 1-nuclear factor erythroid 2-related factor 2-prohibitin 2 pathway, leading to mitochondrial dysfunction and cognitive decline [30]. Mitochondrial dysfunction caused by hyperglycemia also accelerates the generation of ROS and further exacerbates neuronal oxidative damage [31]. In addition, excessive production of AGEs induces endoplasmic reticulum stress [32,33], and telomere length shortening [12,19] is associated with cognitive decline. Regarding the observed decline in cognitive function within the low HGI group, we speculate that it may be related to stress hyperglycemia [14,15]. Low HGI may be associated with higher insulin resistance and gluconeogenesis caused by counterregulatory hormones and pro-inflammatory cytokines in stress hyperglycemia, and the increase of FPG [14,15]. Frequent changes in glucose levels can hinder the supply of glucose to brain tissue, potentially disrupting cognitive processes [34]. The hippocampus is an important region for memory formation [35]. Reducing scores for immediate memory and delayed recall were found to be associated with several cortical and subcortical sites [36]. These areas include the parahippocampal region, posterior cingulate gyrus, right thalamus, and right hippocampus [36]. An animal study found that adult male mice experiencing chronic social failure stress developed hyperglycemia, which was associated with hippocampus-related spatial memory impairment [37]. Our study highlights the relationship of changes in HGI levels with cognitive function, and the potential benefits of early intervention need further investigation in longitudinal studies.

The association of reduced HGI with cognitive decline (Z-score and CERAD) was found in females. The reason for this may be that older women experience menopause, estrogen changes, which may be related to cognitive changes [38,39]. When the menopausal estrogen is reduced, inflammatory markers (interleukin-6, interleukin-1, and tumor necrosis factor-α) are also increased, and this inflammatory state may result in further aggravation of cognitive impairment [40]. The observed association between high HGI and lower DST scores in males may be due to mechanisms such as oxidative stress or mitochondrial damage caused by high HGI [28–30]. The observed lower cognitive level in the low HGI group among participants not eligible for antidiabetic drugs may be primarily driven by CERAD. However, the specific mechanism of HGI differences in cognitive function among different sex and antidiabetic drug-used participants was not available in this study, and further experimental and animal studies may help to explain our results.

Our study presents, varying associations between different HGI and cognitive functions in different domains in the elderly. Our findings suggest that HGI provides simple and easily available markers to detect cognitive deterioration in older adults. From a clinical perspective, changes in HGI levels may serve as an early warning indicator for cognitive decline, monitoring of HGI provides an effective tool for identifying older adults who may develop cognitive dysfunction. Physicians can timely identify patients with poor glycemic control by regular examination of HGI, and take corresponding interventions, such as diet modification, drug therapy, or lifestyle intervention, to improve metabolic status, which may slow down the decline of cognitive function.

We must acknowledge that several limitations remain. First of all, the cross-sectional nature of this study restricts our ability to reveal temporal or causal inferences related to the observed associations. Secondly, due to the limited cognitive function tests, this study only focused on the NHANES data from 2011 to 2014. Thirdly, the exclusion of approximately 54.9% of eligible participants due to incomplete FPG data may introduce selection bias. Fourthly, there may still be some confounders that could not be considered although adequate controls for potential confounding factors were implemented, which may introduce a degree of bias. Finally, the direction of HGI changes, different study populations may be related to different functions of HGI-induced cognitive function changes. The underlying mechanisms need to be further verified.

## 5. Conclusion

In the present study, lower HGI or higher HGI may be associated with cognitive decline among older adults in the U.S. In addition, lower HGI may associated with worse learning and memory dimensions, while higher HGI may be associated with impaired executive function. While the associations differ among populations with different sex and antidiabetic drugs used, our results highlight the necessity for continued investigations on the underlying mechanism of HGI and cognitive function. The cross-sectional nature of this study restricts our ability to reveal temporal or causal inferences related to the observed associations. Future studies need to further validate our findings.

## Supporting information

S1 Fig The linearity, normality and homoscedasticity of residuals, and residual plots (Z-score). A. linearity; B. normality; C. homoscedasticity; D. residual plots.
(PDF)

S2 Fig The linearity, normality and homoscedasticity of residuals, and residual plots (CREAD score). A. linearity; B. normality; C. homoscedasticity; D. residual plots.
(PDF)

S3 Fig The linearity, normality and homoscedasticity of residuals, and residual plots (AFT score). A. linearity; B. normality; C. homoscedasticity; D. residual plots.
(PDF)

S4 Fig The linearity, normality and homoscedasticity of residuals, and residual plots (DSST score). A. linearity; B. normality; C. homoscedasticity; D. residual plots.
(PDF)

S1 File Supplementary Tables. (DOCX)
