# Supplementary figures and images for: Association between hemoglobin glycation index and cognitive function: Evidence in the elderly
Source: PLoS One. 2026 May 15;21(5):e0338613. doi: 10.1371/journal.pone.0338613 (PMC13178853; doi:10.1371/journal.pone.0338613)

A

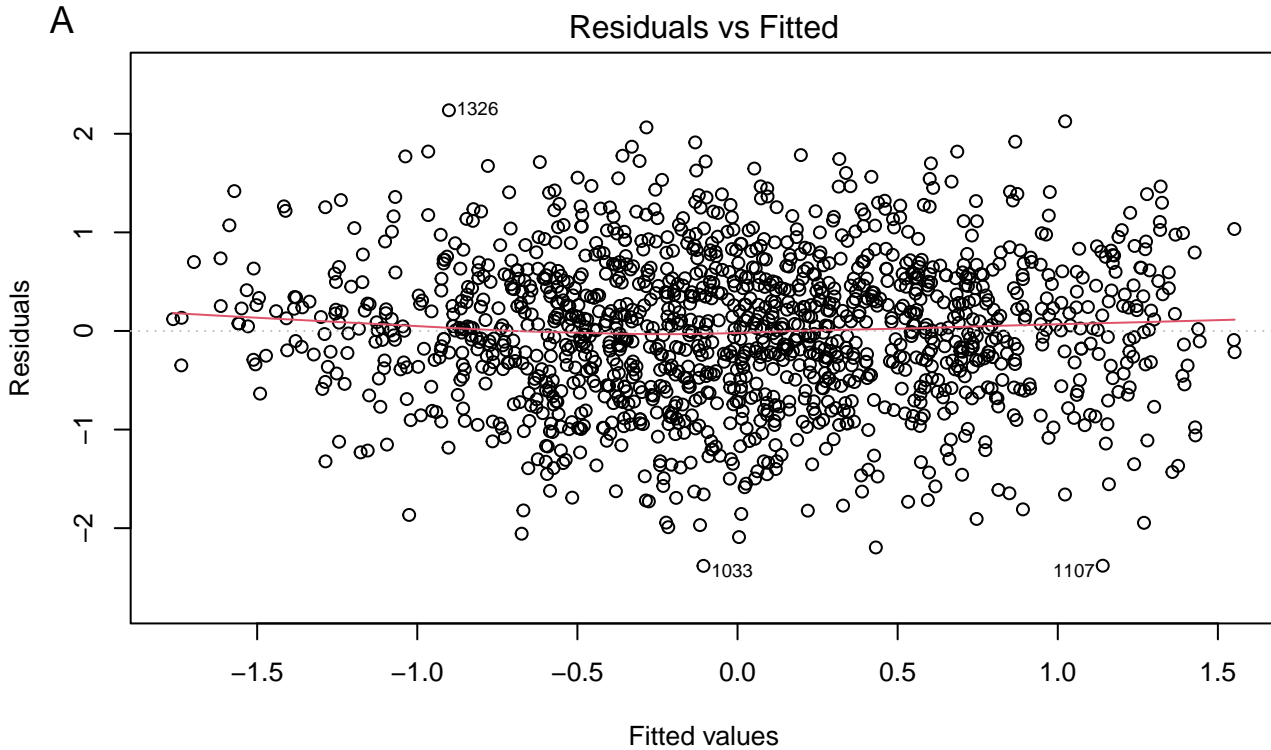

B

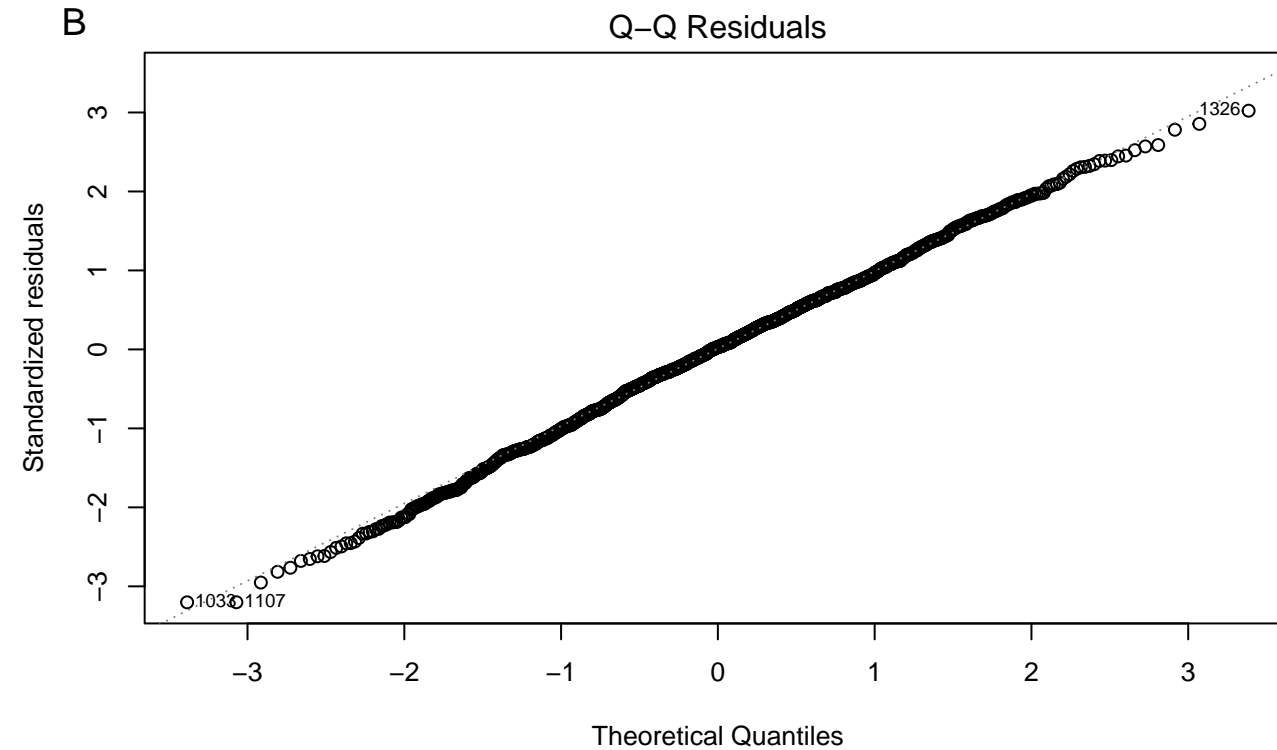

C

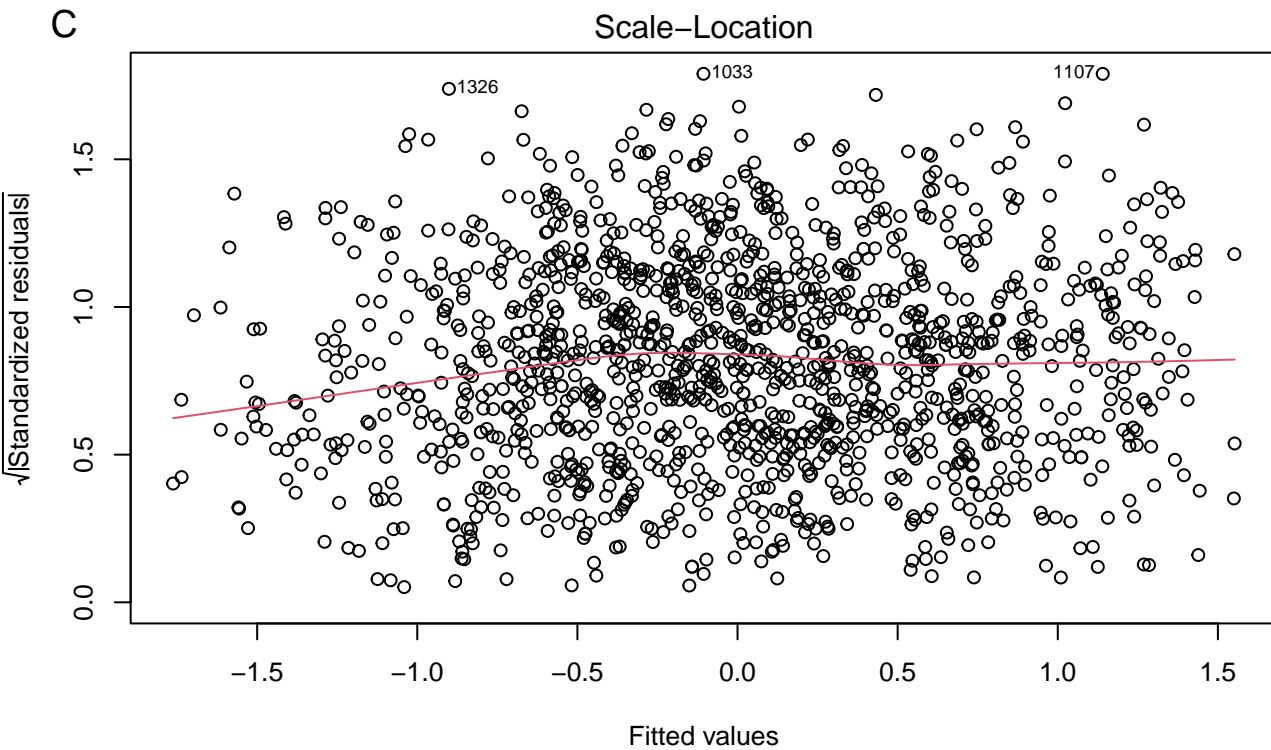

D

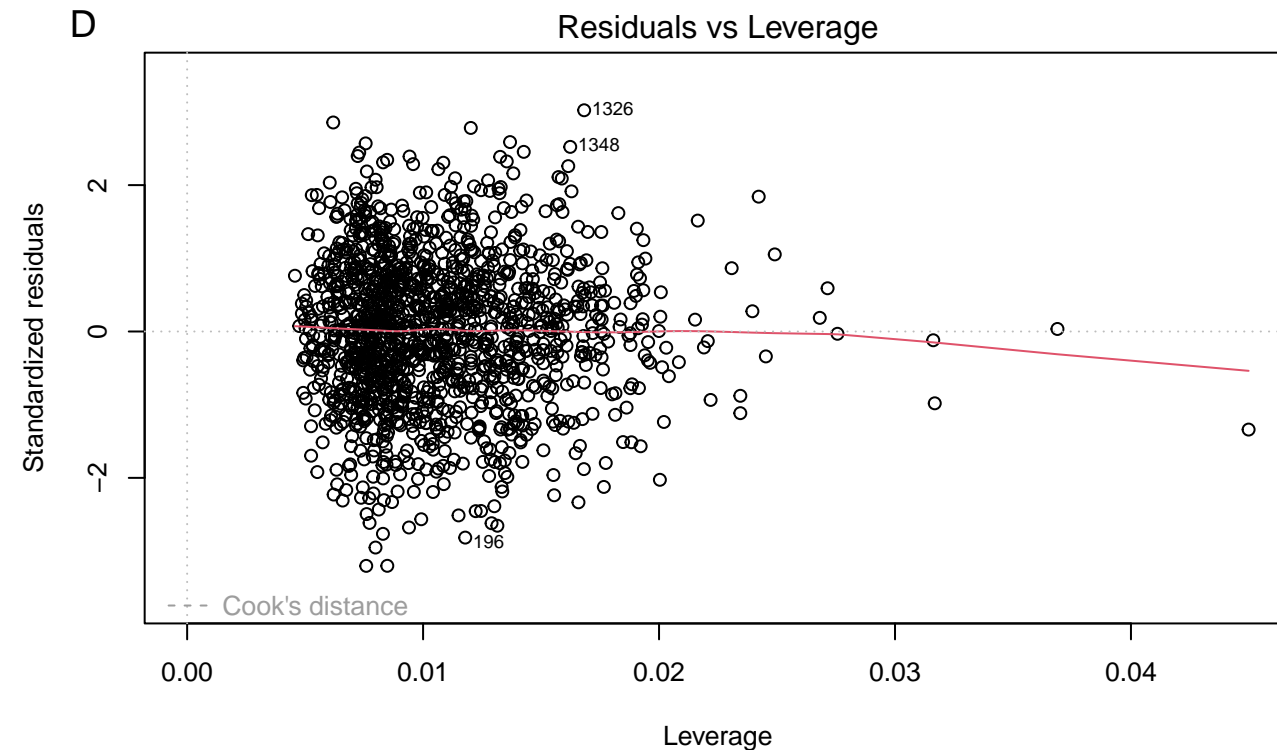

Supplement: S1 Fig — A. linearity; B. normality; C. homoscedasticity; D. residual plots. (PDF) [file pone.0338613.s001.pdf]

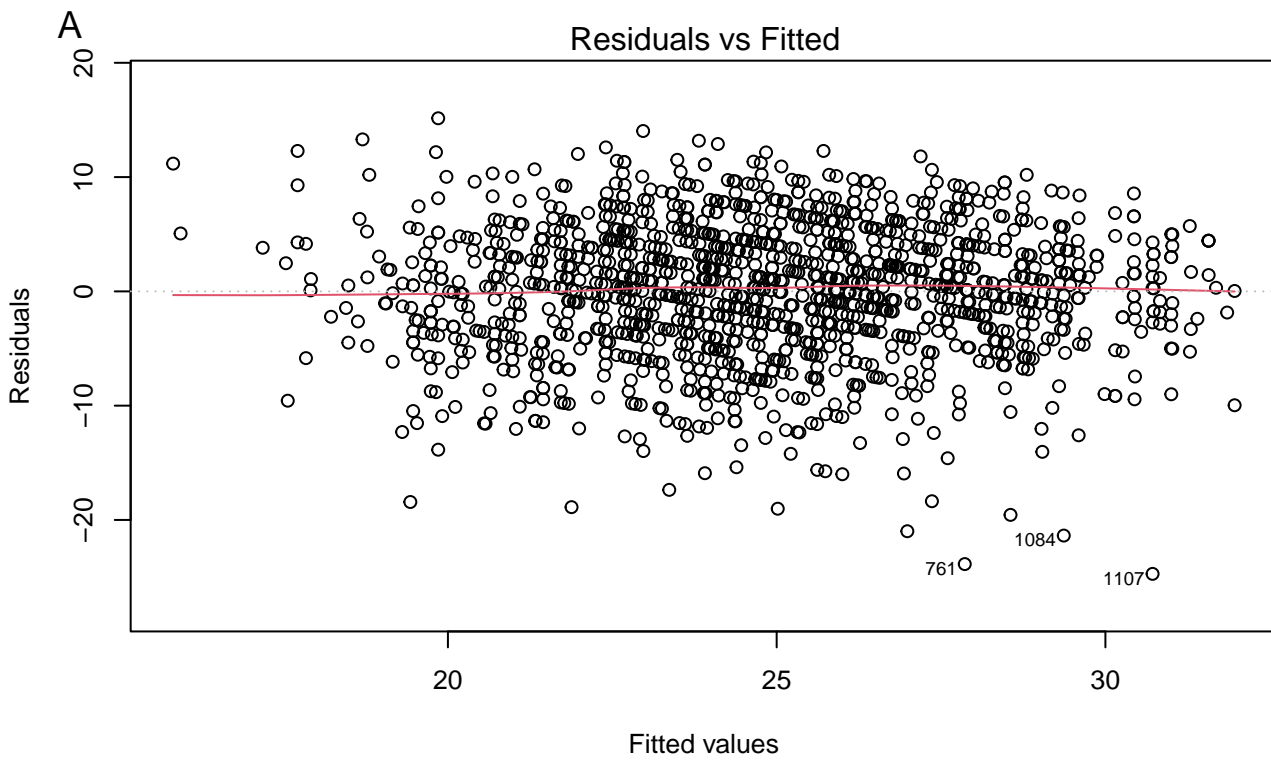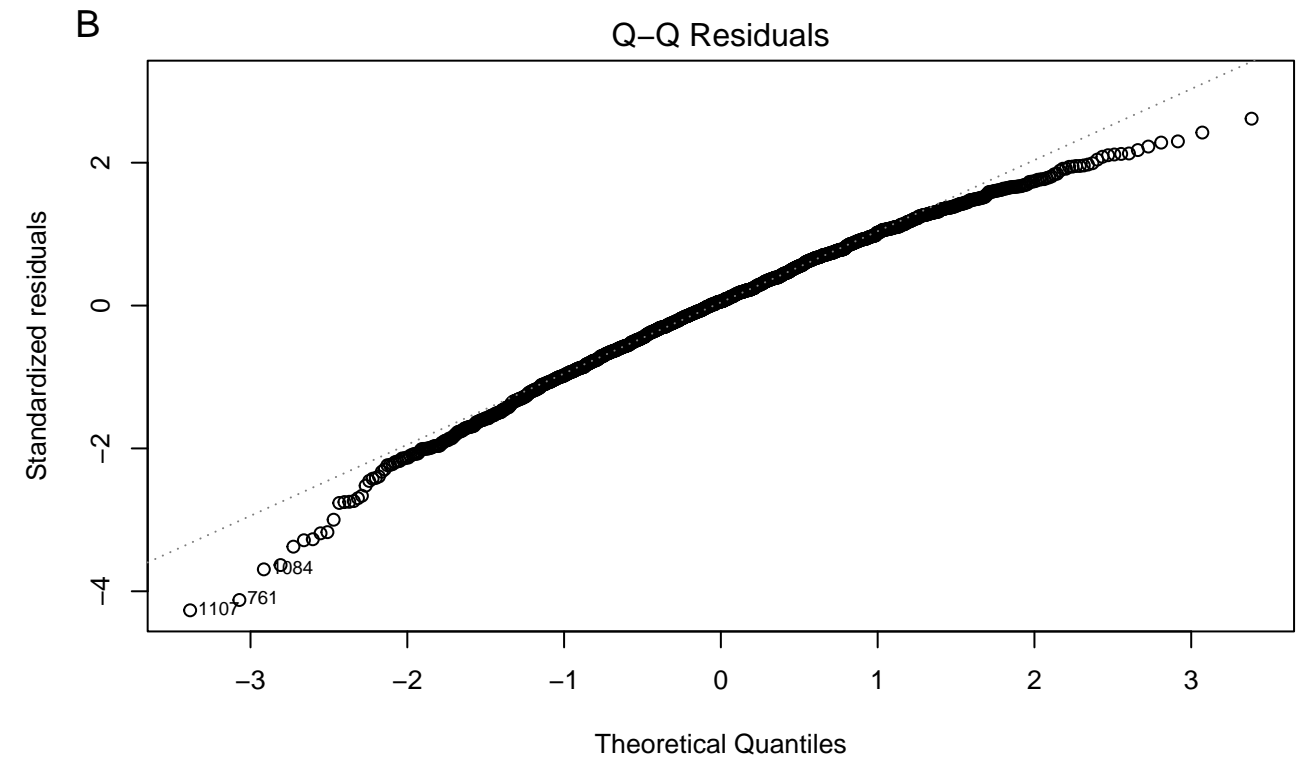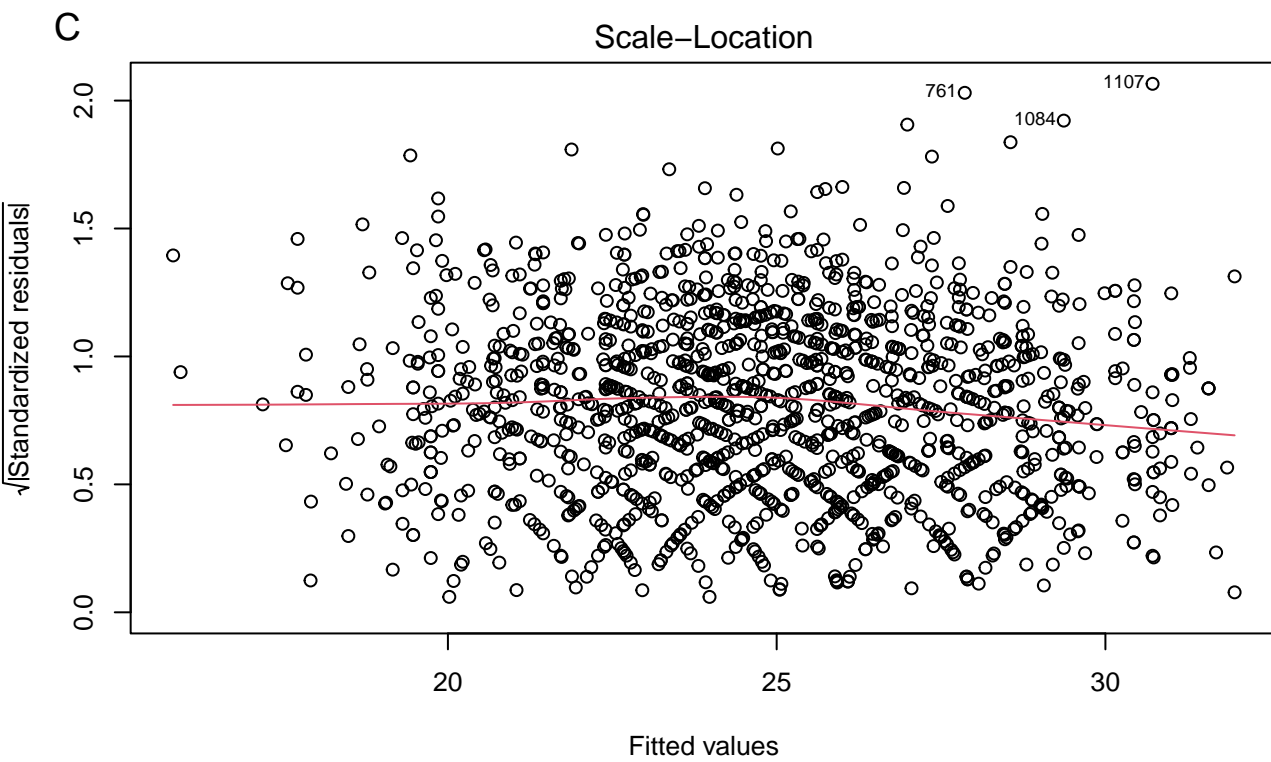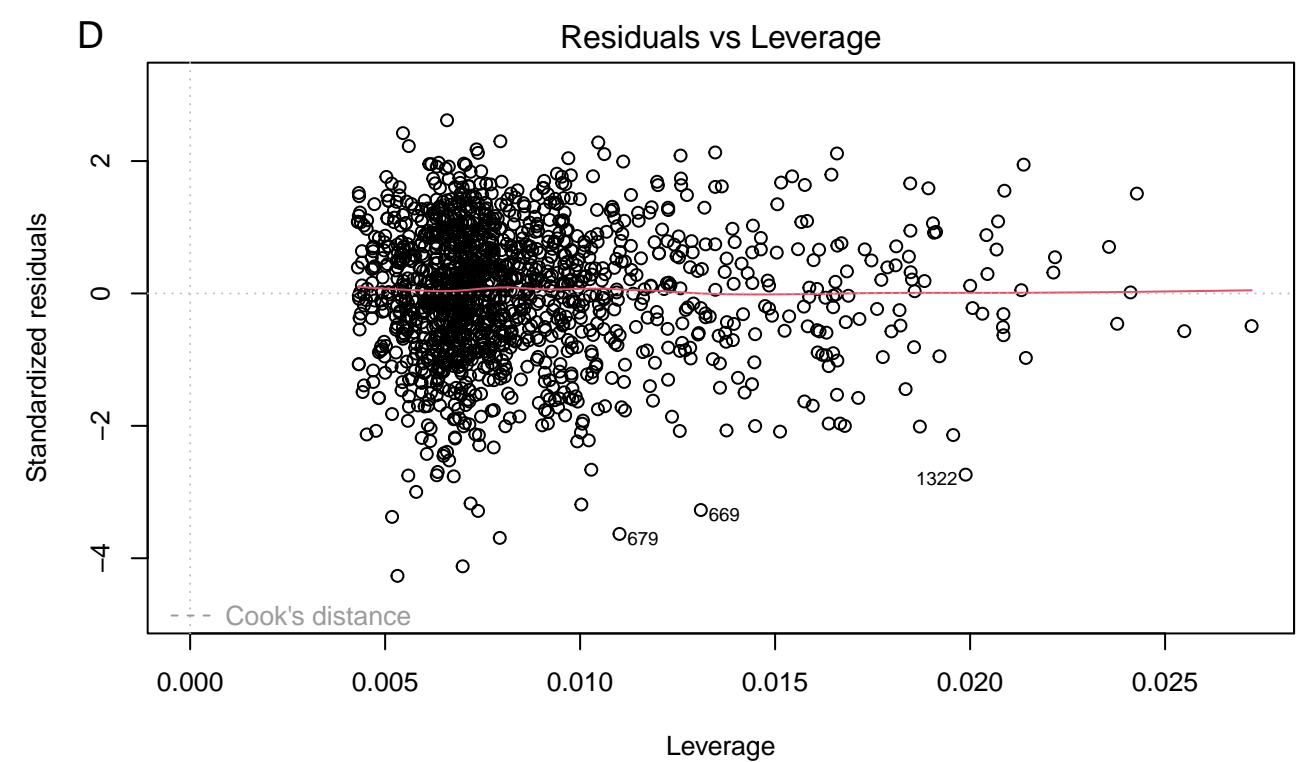

Supplement: S2 Fig — A. linearity; B. normality; C. homoscedasticity; D. residual plots. (PDF) [file pone.0338613.s002.pdf]

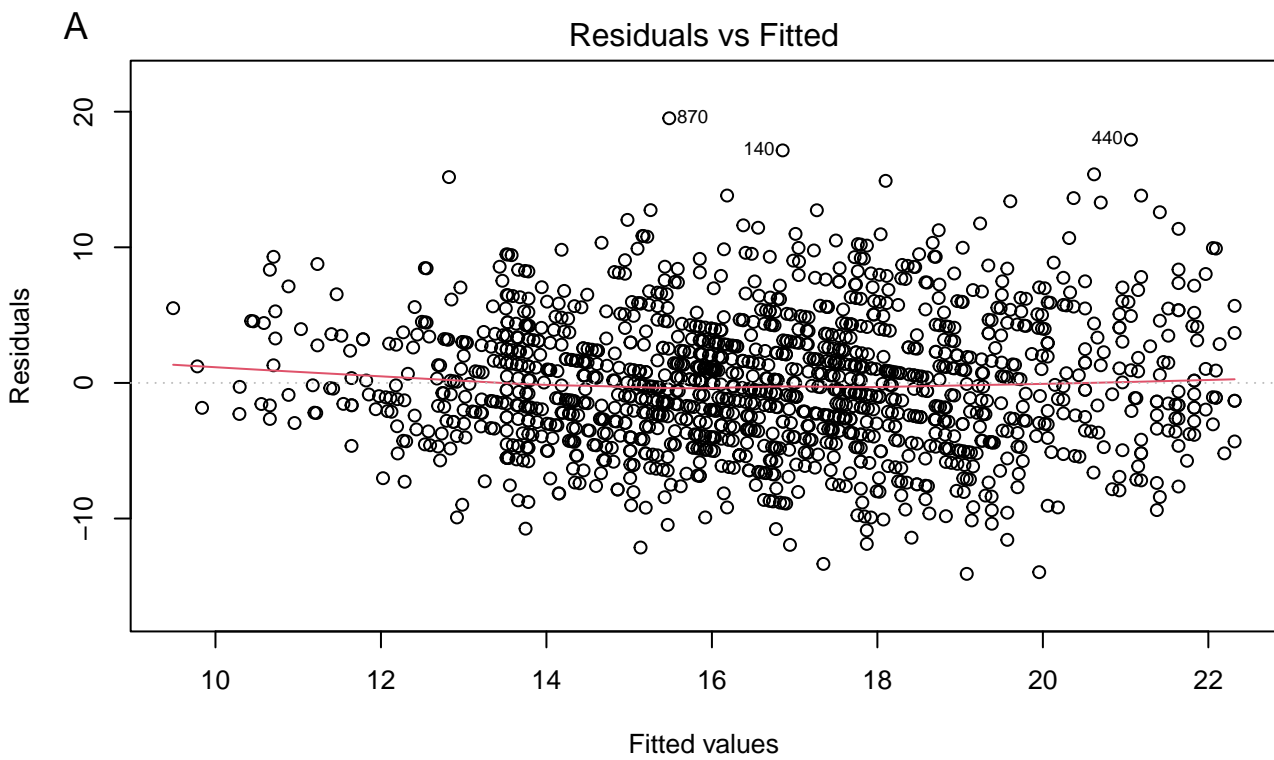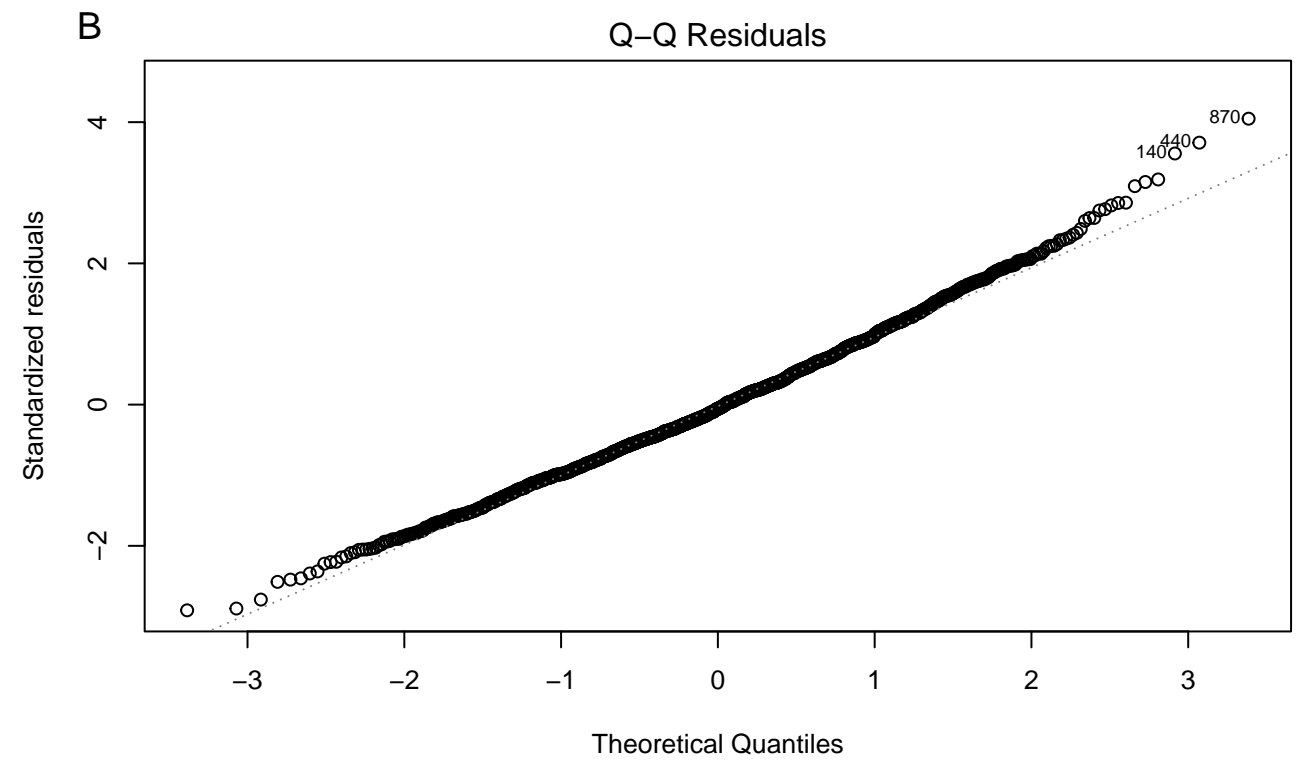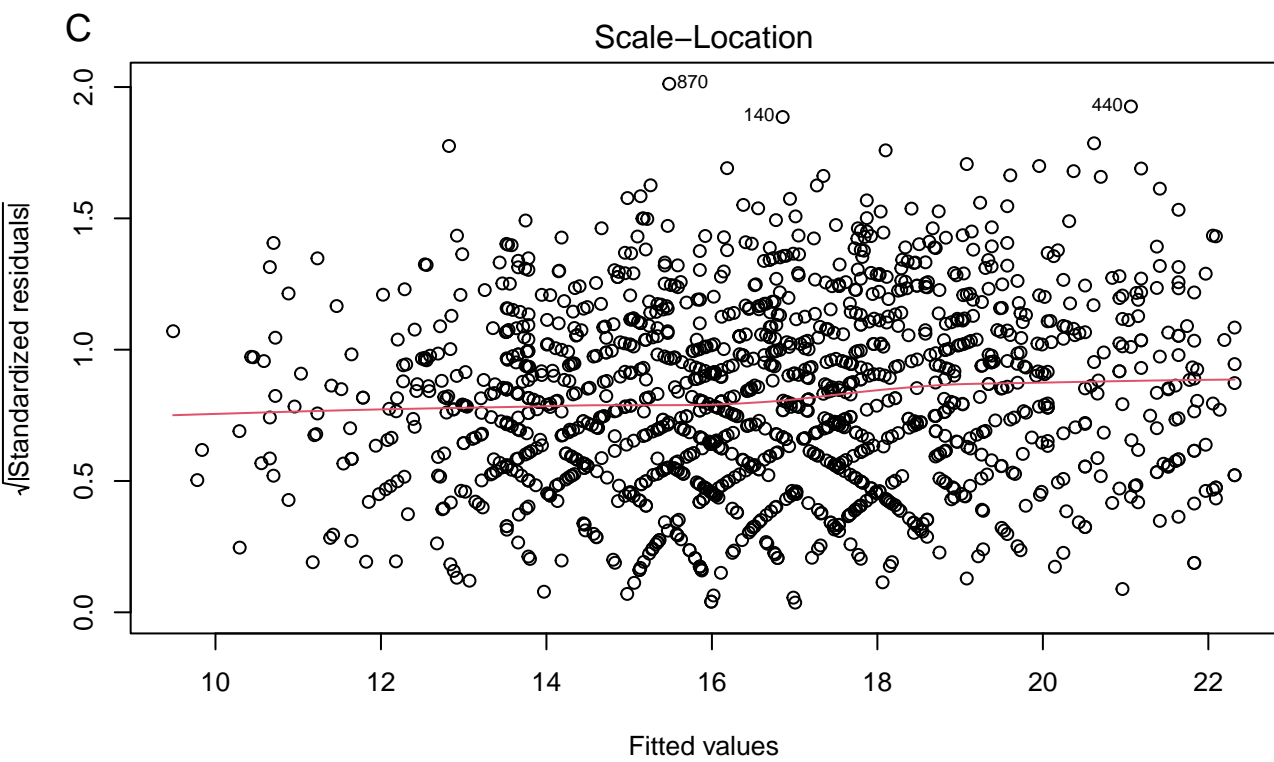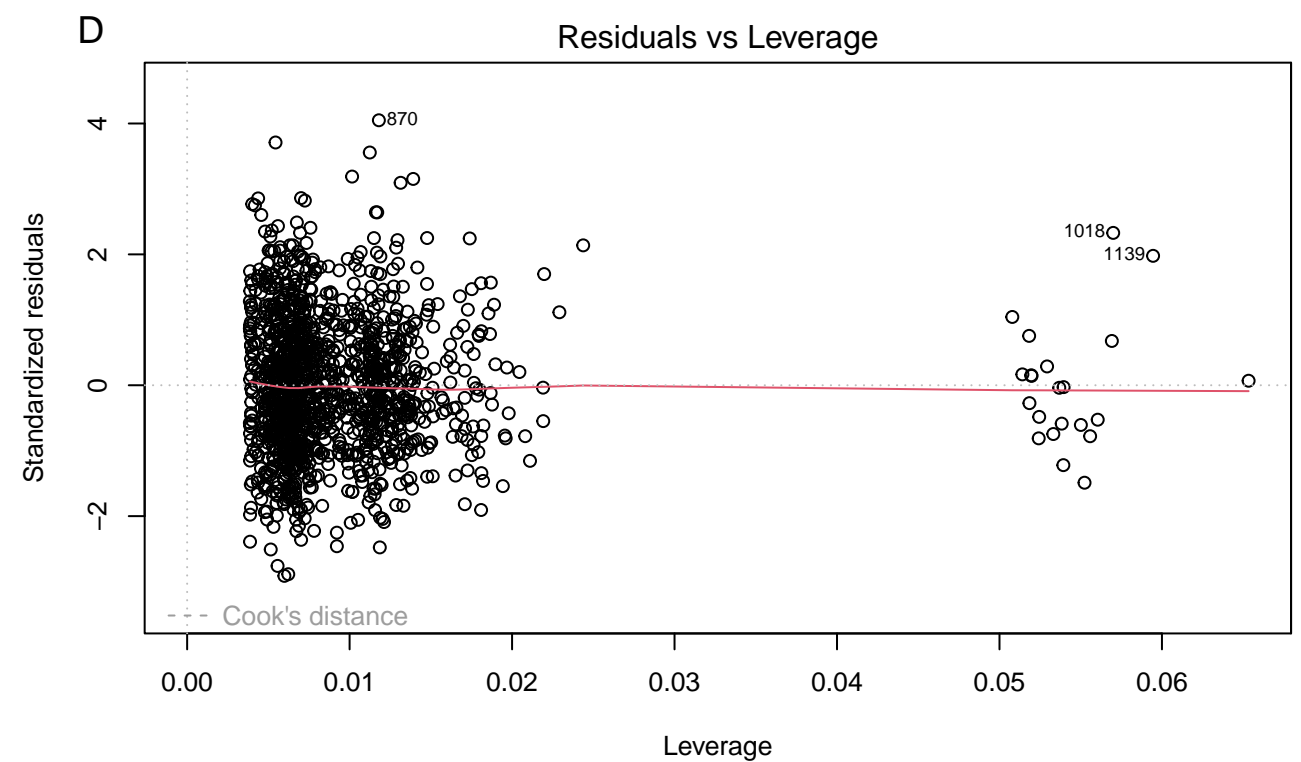

Supplement: S3 Fig — A. linearity; B. normality; C. homoscedasticity; D. residual plots. (PDF) [file pone.0338613.s003.pdf]

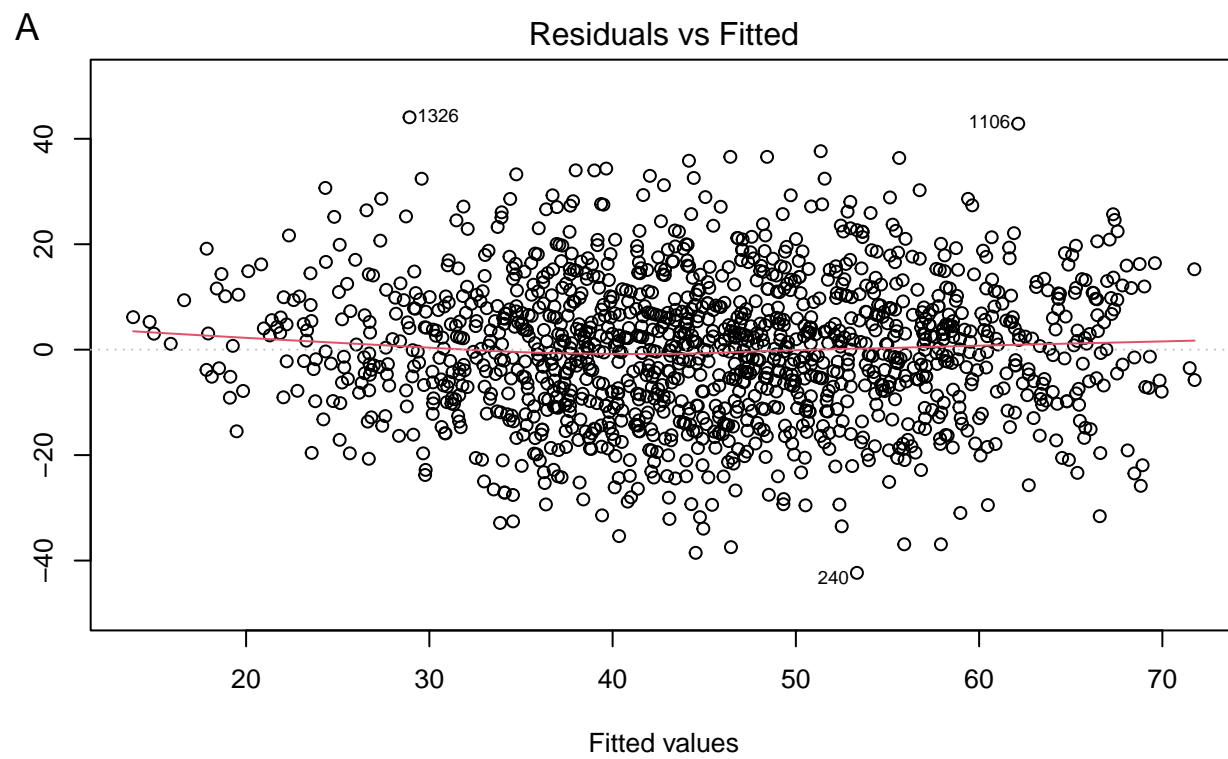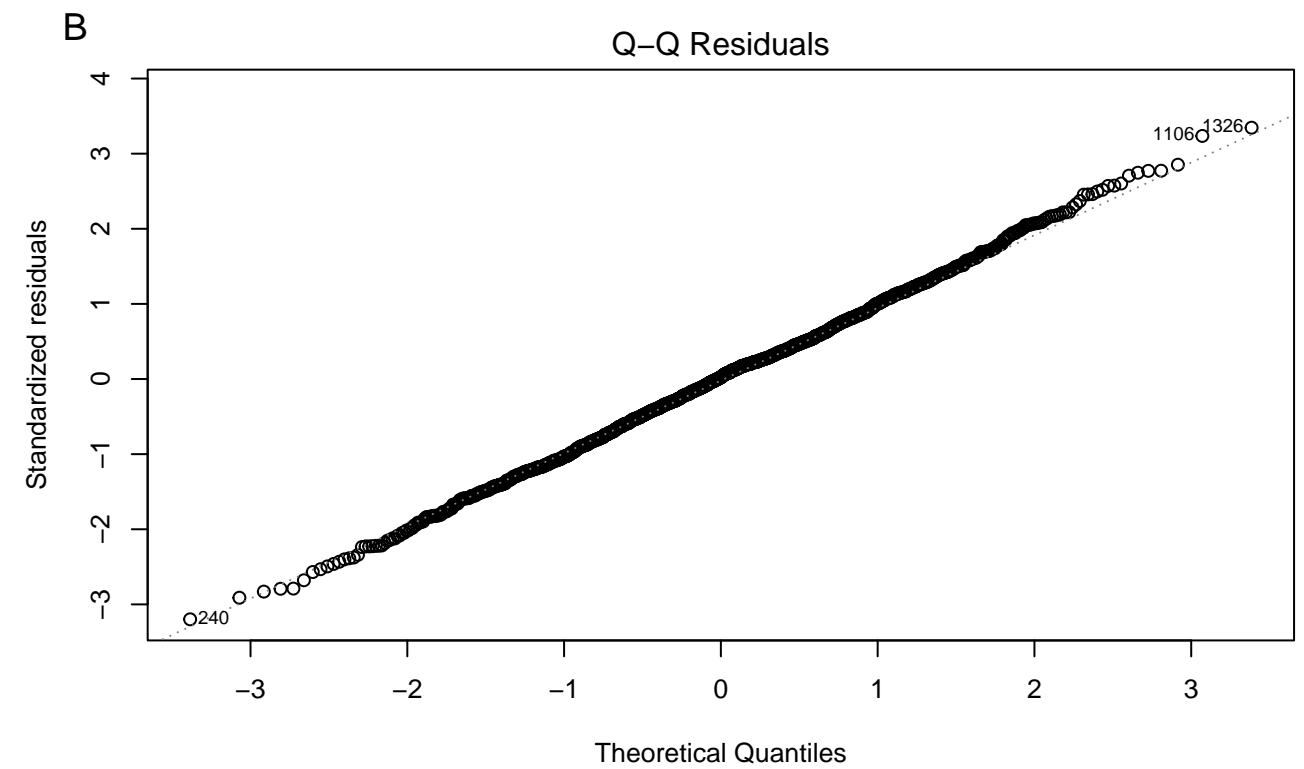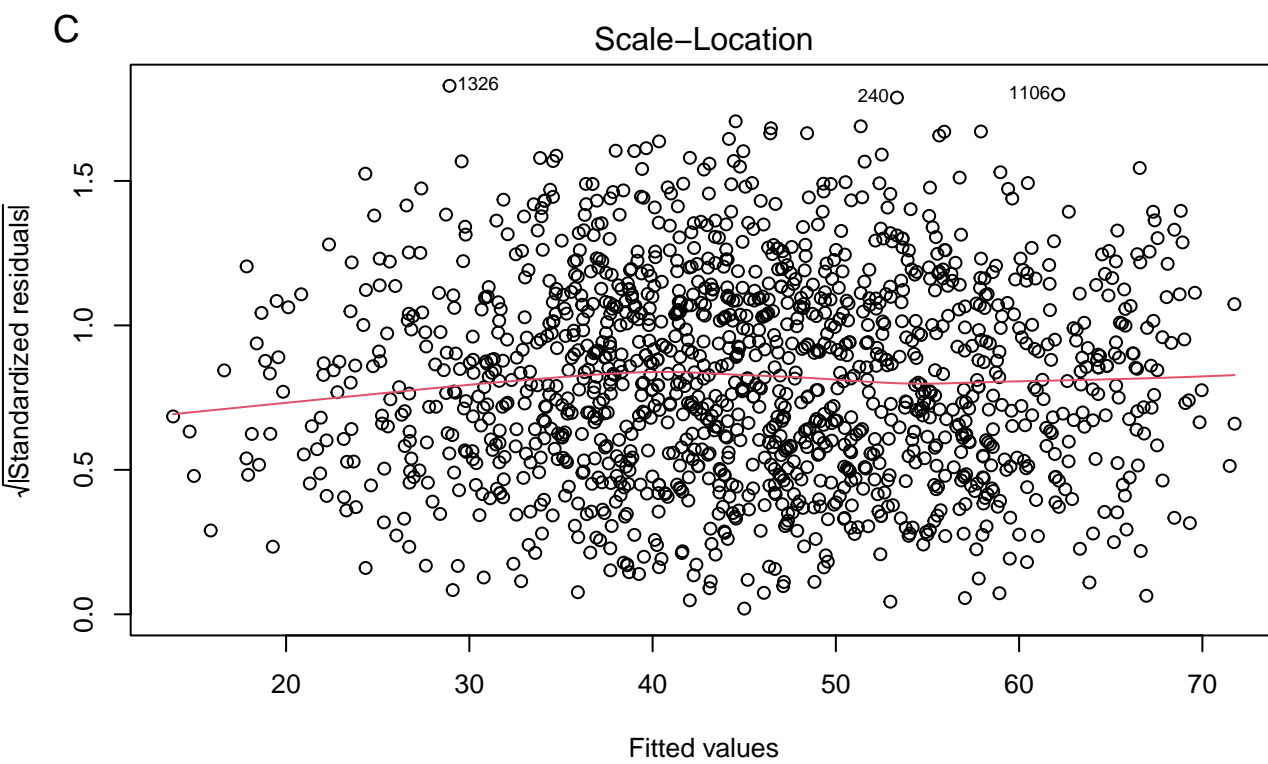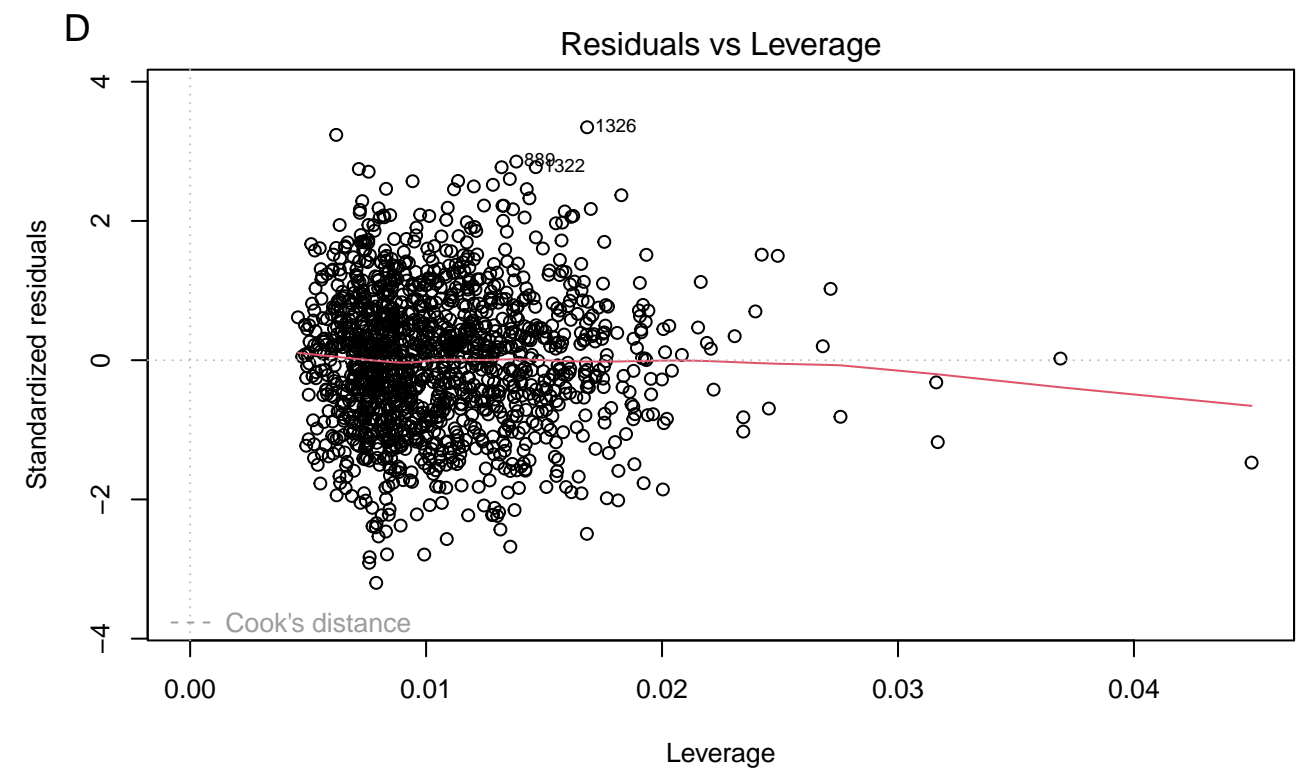

Supplement: S4 Fig — A. linearity; B. normality; C. homoscedasticity; D. residual plots. (PDF) [file pone.0338613.s004.pdf]
