# Supplementary material for: Association between hemoglobin glycation index and cognitive function: Evidence in the elderly
Source: PLoS One. 2026 May 15;21(5):e0338613. doi: 10.1371/journal.pone.0338613 (PMC13178853; doi:10.1371/journal.pone.0338613)
Supplement: S1 File — (DOCX) [file pone.0338613.s005.docx]

Table S1. The comparing baseline characteristics between included and excluded cohorts

| Variables | Total (n=3632) | Include cohort (n=1637) | Exclude cohort (n=1995) | Statistics | *P* |
| --- | --- | --- | --- | --- | --- |
| Age, years, Mean (±S.E) | 69.65 (0.20) | 69.62 (0.26) | 69.67 (0.25) | t=0.145 | 0.885 |
| Gender, n (%) |  |  |  | χ²=1.360 | 0.252 |
| Male | 1760 (45.07) | 813 (46.12) | 947 (44.06) |  |  |
| Female | 1872 (54.93) | 824 (53.88) | 1048 (55.94) |  |  |
| Race, n (%) |  |  |  | χ²=2.724 | 0.086 |
| Non-Hispanic White | 1648 (77.35) | 785 (78.76) | 863 (75.99) |  |  |
| Non-Hispanic Black | 871 (9.05) | 349 (7.63) | 522 (10.43) |  |  |
| Mexican American | 336 (3.84) | 150 (3.61) | 186 (4.06) |  |  |
| Other Race | 777 (9.76) | 353 (10.00) | 424 (9.52) |  |  |
| PIR, n (%) |  |  |  | χ²=0.793 | 0.380 |
| <1 | 652 (10.89) | 283 (10.37) | 369 (11.40) |  |  |
| ≥1 | 2634 (89.11) | 1202 (89.63) | 1432 (88.60) |  |  |
| Marriage, n (%) |  |  |  | χ²=11.169 | 0.002 |
| Married/Living with partner | 2021 (63.24) | 987 (67.44) | 1034 (59.17) |  |  |
| Single/Separated/Divorced/Widowed | 1604 (36.76) | 648 (32.56) | 956 (40.83) |  |  |
| Education, n (%) |  |  |  | χ²=0.626 | 0.435 |
| High School and below | 1910 (41.01) | 867 (42.06) | 1043 (39.99) |  |  |
| Above high school | 1714 (58.99) | 767 (57.94) | 947 (60.01) |  |  |
| PA, n (%) |  |  |  | χ²=1.553 | 0.211 |
| <450 | 427 (11.11) | 179 (9.79) | 248 (12.39) |  |  |
| 450-750 | 318 (8.48) | 148 (8.68) | 170 (8.28) |  |  |
| ≥750 | 1473 (44.24) | 701 (45.87) | 772 (42.65) |  |  |
| Unknown | 1414 (36.17) | 609 (35.66) | 805 (36.67) |  |  |
| Smoking, n (%) |  |  |  | χ²=0.310 | 0.710 |
| Never | 1815 (49.90) | 822 (48.90) | 993 (50.88) |  |  |
| Ever | 1347 (38.84) | 609 (39.95) | 738 (37.77) |  |  |
| Now | 465 (11.25) | 204 (11.15) | 261 (11.36) |  |  |
| Hypertension, n (%) |  |  |  | χ²=9.665 | 0.004 |
| No | 600 (17.82) | 282 (20.30) | 318 (15.41) |  |  |
| Yes | 3032 (82.18) | 1355 (79.70) | 1677 (84.59) |  |  |
| Antidiabetic drug, n (%) |  |  |  | χ² = 0.038 | 0.846 |
| No | 2787 (80.71) | 1260 (80.47) | 1527 (80.95) |  |  |
| Yes | 844 (19.29) | 377 (19.53) | 467 (19.05) |  |  |
| Dyslipidemia, n (%) |  |  |  | χ² = 0.007 | 0.933 |
| No | 629 (14.82) | 243 (14.90) | 386 (14.74) |  |  |
| Yes | 3002 (85.18) | 1394 (85.10) | 1608 (85.26) |  |  |
| Heavy Drinking, n (%) |  |  |  | χ² = 0.317 | 0.726 |
| No | 2149 (64.60) | 1031 (64.61) | 1118 (64.60) |  |  |
| Yes | 494 (15.58) | 232 (16.13) | 262 (15.04) |  |  |
| Unknown | 989 (19.82) | 374 (19.26) | 615 (20.36) |  |  |
| BMI, n (%) |  |  |  | χ² = 1.507 | 0.228 |
| <25 | 957 (26.92) | 459 (28.12) | 498 (25.73) |  |  |
| ≥25 | 2438 (73.08) | 1155 (71.88) | 1283 (74.27) |  |  |
| Depression, n (%) |  |  |  | χ² = 0.405 | 0.529 |
| No | 2914 (92.41) | 1374 (92.02) | 1540 (92.78) |  |  |
| Yes | 304 (7.59) | 147 (7.98) | 157 (7.22) |  |  |
| CKD, n (%) |  |  |  | χ² = 0.484 | 0.492 |
| No | 2185 (73.10) | 1095 (73.94) | 1090 (72.22) |  |  |
| Yes | 952 (26.90) | 469 (26.06) | 483 (27.78) |  |  |
| Stroke, n (%) |  |  |  | χ² = 0.446 | 0.509 |
| No | 3317 (92.33) | 1497 (91.99) | 1820 (92.65) |  |  |
| Yes | 315 (7.67) | 140 (8.01) | 175 (7.35) |  |  |
| Antiparkinson agents, n (%) |  |  |  | χ² = 0.041 | 0.840 |
| No | 3576 (98.28) | 1612 (98.22) | 1964 (98.34) |  |  |
| Yes | 56 (1.72) | 25 (1.78) | 31 (1.66) |  |  |
| Anxiolytics sedatives hypnotics, n (%) |  |  |  | χ² = 0.177 | 0.676 |
| No | 3408 (93.34) | 1540 (93.65) | 1868 (93.04) |  |  |
| Yes | 224 (6.66) | 97 (6.35) | 127 (6.96) |  |  |
| Psychotherapeutic agents, n (%) |  |  |  | χ² = 0.194 | 0.663 |
| No | 3063 (81.24) | 1364 (80.75) | 1699 (81.71) |  |  |
| Yes | 569 (18.76) | 273 (19.25) | 296 (18.29) |  |  |
| Total energy, kcal, Mean (±S.E) | 1888.91 (20.49) | 1876.00 (31.07) | 1901.70 (28.64) | t = 0.592 | 0.558 |
| Carbohydrate, gm, Mean (±S.E) | 226.78 (2.99) | 221.59 (3.97) | 231.92 (3.79) | t = 2.099 | 0.044 |
| Protein, gm, Mean (±S.E) | 73.05 (0.76) | 73.82 (1.50) | 72.29 (1.15) | t = -0.696 | 0.492 |
| Total fat, gm, Mean (±S.E) | 73.92 (0.86) | 74.38 (1.62) | 73.47 (1.41) | t = -0.362 | 0.720 |
| Total sugars, gm, Mean (±S.E) | 98.06 (1.53) | 94.79 (1.93) | 101.31 (1.94) | t = 2.717 | 0.011 |
| Diabetes, n (%) |  |  |  | χ² = 7.016 | 0.012 |
| No | 2413 (72.42) | 1012 (68.77) | 1401 (75.96) |  |  |
| Yes | 1218 (27.58) | 625 (31.23) | 593 (24.04) |  |  |
| Z-score, Mean (±S.E) | 0.36 (0.03) | 0.35 (0.05) | 0.37 (0.03) | t=0.406 | 0.688 |
| CERAD, Mean (±S.E) | 25.64 (0.29) | 25.96 (0.38) | 25.32 (0.24) | t=-2.317 | 0.027 |
| AFT, Mean (±S.E) | 17.88 (0.18) | 17.89 (0.22) | 17.87 (0.22) | t=-0.081 | 0.936 |
| DSST, Mean (±S.E) | 51.83 (0.55) | 51.32 (0.77) | 52.34 (0.63) | t=1.187 | 0.244 |
| HbA1c, %, Mean (±S.E) | 5.96 (0.02) | 5.98 (0.05) | 5.94 (0.02) | t=-0.659 | 0.515 |

Mean ± standard error or frequencies (percentage) was used in the data description. HGI, hemoglobin glycation index; PIR, poverty income ratio; PA, physical activity; BMI, body mass index; CKD, chronic kidney disease; CERAD, Consortium to Establish a Registry for Alzheimer's disease; AFT, Animal Fluency Test; DSST, Digit Symbol Substitution Test; HbA1c, glycated hemoglobin; FBG, fasting plasma glucose. P-values were obtained using the weighted one-way analysis of variance or the Rao-Scott Chi-square test, respectively.

Table S2. Missing data

| Variables | n | Percentage | Method |
| --- | --- | --- | --- |
| Marriage | 1 | 0.07% | Multiple imputation |
| Education | 2 | 0.14% | Multiple imputation |
| Smoking | 2 | 0.14% | Multiple imputation |
| BMI | 15 | 1.07% | Multiple imputation |
| Depression | 21 | 1.49% | Multiple imputation |
| CKD | 42 | 2.99% | Multiple imputation |
| Total energy | 104 | 7.4% | Multiple imputation |
| Carbohydrate | 104 | 7.4% | Multiple imputation |
| Protein | 104 | 7.4% | Multiple imputation |
| Total fat | 104 | 7.4% | Multiple imputation |
| Total sugars | 104 | 7.4% | Multiple imputation |
| PIR | 124 | 8.82% | Multiple imputation |

BMI, body mass index; CKD, chronic kidney disease.

Table S3. Sensitivity analysis before and after data interpolation (n = 1,406)

| Variables | Before imputation | After imputation | *P* |
| --- | --- | --- | --- |
| Marriage, n (%) |  |  | 0.329 |
| Married/living with partner | 873 (68.47) | 874 (68.48) |  |
| Living alone | 532 (31.53) | 532 (31.52) |  |
| Education, n (%) |  |  | 0.169 |
| High school and below | 700 (39.50) | 702 (39.53) |  |
| Above high school | 704 (60.50) | 704 (60.47) |  |
| Smoking, n (%) |  |  | 0.157 |
| Never | 697 (48.99) | 699 (49.01) |  |
| Former | 539 (40.54) | 539 (40.52) |  |
| Current | 168 (10.47) | 168 (10.47) |  |
| BMI, (kg/m^2^) | 29.19 ± 0.34 | 29.18 ± 0.34 | 0.754 |
| Depression, n (%) |  |  | 0.710 |
| No | 1253 (91.99) | 1273 (91.95) |  |
| Yes | 132 (8.01) | 133 (8.05) |  |
| CKD, n (%) |  |  | 0.659 |
| No | 973 (75.80) | 1008 (75.66) |  |
| Yes | 391 (24.20) | 398 (24.34) |  |
| Total energy (kcal) | 1891.16 ± 31.82 | 1887.92 ±30.89 | 0.053 |
| Carbohydrate (gm) | 222.87 ± 4.13 | 222.63 ± 4.00 | 0.176 |
| Protein (gm) | 74.32 ± 1.58 | 74.16 ± 1.53 | 0.063 |
| Total fat (gm) | 75.34 ± 1.54 | 75.19 ± 1.50 | 0.090 |
| Total sugars (gm) | 94.69 ± 2.07 | 94.66 ± 1.99 | 0.739 |
| PIR, n (%) |  |  | 0.977 |
| <1 | 215 (8.69) | 226 (8.69) |  |
| ≥1 | 1067 (91.31) | 1180 (91.31) |  |

Data were expressed as mean ± standard error or frequencies (percentage). PIR, poverty income ratio; BMI, body mass index; CKD, chronic kidney disease. *P*-values were obtained using weighted one-way analysis of variance or the Rao-Scott Chi-square test.

Table S4. The association between confounding variables and Z-scores (n = 1,406)

| Variables | Model 1 | *P* | Model 2 | *P* |
| --- | --- | --- | --- | --- |
|  | β (95% CI) |  | β (95% CI) |  |
| Age | -0.07 (-0.08, -0.06) | <0.001 | -0.06 (-0.07, -0.05) | <0.001 |
| Gender |  |  |  |  |
| Male | Ref |  | Ref |  |
| Female | 0.23 (0.12, 0.33) | <0.001 | 0.38 (0.28, 0.48) | <0.001 |
| Race |  |  |  |  |
| Non-Hispanic White | Ref |  | Ref |  |
| Non-Hispanic Black | -0.82 (-1.01, -0.63) | <0.001 | -0.74 (-0.90, -0.57) | <0.001 |
| Mexican American | -0.85 (-1.05, -0.66) | <0.001 | -0.70 (-0.89, -0.51) | <0.001 |
| Other race | -0.61 (-0.88, -0.35) | <0.001 | -0.55 (-0.71, -0.39) | <0.001 |
| PIR |  |  |  |  |
| <1 | Ref |  | Ref |  |
| ≥1 | 0.83 (0.61, 1.04) | <0.001 | 0.36 (0.20, 0.52) | <0.001 |
| Marriage |  |  |  |  |
| Married or living with partner | Ref |  |  |  |
| Living alone | -0.28 (-0.44, -0.12) | 0.001 |  |  |
| Education |  |  |  |  |
| High school and below | Ref |  | Ref |  |
| Above high school | 0.88 (0.75, 1.02) | <0.001 | 0.55 (0.45, 0.65) | <0.001 |
| PA |  |  |  |  |
| <450 | Ref |  |  |  |
| 450-750 | 0.10 (-0.19, 0.39) | 0.491 |  |  |
| ≥750 | 0.44 (0.21, 0.67) | 0.001 |  |  |
| Unknown | -0.07 (-0.29, 0.16) | 0.557 |  |  |
| Smoking |  |  |  |  |
| Never | Ref |  | Ref |  |
| Former | -0.11 (-0.28, 0.06) | 0.185 | -0.06 (-0.19, 0.07) | 0.382 |
| Current | -0.36 (-0.56, -0.16) | 0.001 | -0.20 (-0.36, -0.04) | 0.018 |
| Hypertension |  |  |  |  |
| No | Ref |  |  |  |
| Yes | -0.46 (-0.63, -0.29) | <0.001 |  |  |
| Antidiabetic drug |  |  |  |  |
| No | Ref |  |  |  |
| Yes | -0.53 (-0.74, -0.31) | <0.001 |  |  |
| Dyslipidemia |  |  |  |  |
| No | Ref |  |  |  |
| Yes | -0.14 (-0.33, 0.05) | 0.136 |  |  |
| Heavy drinking |  |  |  |  |
| No | Ref |  |  |  |
| Yes | 0.20 (0.00, 0.39) | 0.047 |  |  |
| Unknown | -0.38 (-0.52, -0.23) | <0.001 |  |  |
| BMI |  |  |  |  |
| <25 | Ref |  |  |  |
| ≥25 | 0.03 (-0.15, 0.21) | 0.746 |  |  |
| Depression |  |  |  |  |
| No | Ref |  |  |  |
| Yes | -0.39 (-0.63, -0.16) | 0.002 |  |  |
| CKD |  |  |  |  |
| No | Ref |  | Ref |  |
| Yes | -0.59 (-0.78, -0.40) | <0.001 | -0.18 (-0.29, -0.07) | 0.003 |
| Stroke |  |  |  |  |
| No | Ref |  |  |  |
| Yes | -0.38 (-0.68, -0.08) | 0.015 |  |  |
| Antiparkinson agents |  |  |  |  |
| No | Ref |  |  |  |
| Yes | -0.34 (-0.80, 0.11) | 0.135 |  |  |
| Anxiolytics sedatives hypnotics |  |  |  |  |
| No | Ref |  |  |  |
| Yes | -0.23 (-0.59, 0.13) | 0.201 |  |  |
| Psychotherapeutic agents |  |  |  |  |
| No | Ref |  | Ref |  |
| Yes | -0.26 (-0.42, -0.10) | 0.003 | -0.31 (-0.46, -0.16) | <0.001 |
| Total energy | 0.00 (0.00, 0.00) | <0.001 | 0.00 (0.00, 0.00) | 0.004 |
| Carbohydrate | 0.00 (0.00, 0.00) | 0.009 |  |  |
| Protein | 0.00 (0.00, 0.01) | <0.001 |  |  |
| Total fat | 0.00 (0.00, 0.01) | <0.001 |  |  |
| Total sugars | 0.00 (-0.00, 0.00) | 0.135 |  |  |

OR, odds ratio; CI, confidence intervals, Ref., reference; PIR, poverty income ratio; PA, physical activity; BMI, body mass index; CKD, chronic kidney disease.

Model 1 was a weighted univariate linear regression model.

Model 2 was a weighted multivariable linear regression model following backward stepwise regression analysis.

Table S5. The association between confounding variables and CERAD (n = 1,406)

| Variables | Model 1 | *P* | Model 2 | *P* |
| --- | --- | --- | --- | --- |
|  | β (95% CI) |  | β (95% CI) |  |
| Age | -0.33 (-0.40, 0.26) | <0.001 | -0.29 (-0.36, 0.21) | <0.001 |
| Gender |  |  |  |  |
| Male | Ref |  | Ref |  |
| Female | 1.71 (0.89, 2.54) | <0.001 | 1.98 (1.25, 2.70) | <0.001 |
| Race |  |  |  |  |
| Non-Hispanic White | Ref |  | Ref |  |
| Non-Hispanic Black | -2.17 (-3.23, 1.10) | <0.001 | -1.96 (-2.95, 0.96) | <0.001 |
| Mexican American | -2.57 (-3.92, 1.23) | 0.001 | -2.13 (-3.32, 0.94) | 0.001 |
| Other race | -2.41 (-3.89, 0.93) | 0.002 | -2.40 (-3.68, 1.12) | 0.001 |
| PIR |  |  |  |  |
| <1 | Ref |  |  |  |
| ≥1 | 2.68 (1.38, 3.99) | <0.001 |  |  |
| Marriage |  |  |  |  |
| Married or living with partner | Ref |  |  |  |
| Living alone | -1.07 (-2.21, 0.08) | 0.067 |  |  |
| Education |  |  |  |  |
| High school and below | Ref |  | Ref |  |
| Above high school | 3.76 (3.02, 4.49) | <0.001 | 2.71 (1.84, 3.58) | <0.001 |
| PA |  |  |  |  |
| <450 | Ref |  |  |  |
| 450-750 | 0.96 (-0.98, 2.90) | 0.321 |  |  |
| ≥750 | 2.24 (0.32, 4.16) | 0.024 |  |  |
| Unknown | 0.23 (-1.73, 2.20) | 0.811 |  |  |
| Smoking |  |  |  |  |
| Never | Ref |  |  |  |
| Former | -0.89 (-1.91, 0.14) | 0.087 |  |  |
| Current | -1.35 (-2.95, 0.25) | 0.096 |  |  |
| Hypertension |  |  |  |  |
| No | Ref |  | Ref |  |
| Yes | -2.45 (-3.57, 1.33) | <0.001 | -1.17 (-2.20, 0.13) | 0.030 |
| Antidiabetic drug |  |  |  |  |
| No | Ref |  |  |  |
| Yes | -1.98 (-3.38, 0.58) | 0.007 |  |  |
| Dyslipidemia |  |  |  |  |
| No | Ref |  |  |  |
| Yes | -1.03 (-1.75, 0.31) | 0.007 |  |  |
| Heavy drinking |  |  |  |  |
| No | Ref |  |  |  |
| Yes | 0.43 (-1.06, 1.91) | 0.562 |  |  |
| Unknown | -1.41 (-2.35, 0.46) | 0.005 |  |  |
| BMI |  |  |  |  |
| <25 | Ref |  |  |  |
| ≥25 | -0.28 (-1.56, 1.01) | 0.664 |  |  |
| Depression |  |  |  |  |
| No | Ref |  |  |  |
| Yes | -0.82 (-2.40, 0.75) | 0.294 |  |  |
| CKD |  |  |  |  |
| No | Ref |  | Ref |  |
| Yes | -2.65 (-3.49, 1.82) | <0.001 | -0.84 (-1.47, 0.21) | 0.011 |
| Stroke |  |  |  |  |
| No | Ref |  |  |  |
| Yes | -1.22 (-2.79, 0.36) | 0.125 |  |  |
| Antiparkinson agents |  |  |  |  |
| No | Ref |  |  |  |
| Yes | -1.18 (-3.76, 1.41) | 0.360 |  |  |
| Anxiolytics sedatives hypnotics |  |  |  |  |
| No | Ref |  | Ref |  |
| Yes | -1.97 (-3.68, 0.26) | 0.026 | -1.81 (-3.21, 0.41) | 0.013 |
| Psychotherapeutic agents |  |  |  |  |
| No | Ref |  |  |  |
| Yes | -0.96 (-2.16, 0.24) | 0.111 |  |  |
| Total energy | 0.00 (0.00, 0.00) | 0.003 |  |  |
| Carbohydrate | 0.00 (-0.00, 0.01) | 0.093 |  |  |
| Protein | 0.02 (0.00, 0.03) | 0.006 |  |  |
| Total fat | 0.01 (0.01, 0.02) | 0.001 |  |  |
| Total sugars | 0.01 (-0.00, 0.01) | 0.142 |  |  |

CERAD, Consortium to Establish a Registry for Alzheimer's disease; OR, odds ratio; CI, confidence intervals, Ref., reference; PIR, poverty income ratio; PA, physical activity; BMI, body mass index; CKD, chronic kidney disease.

Model 1 was a weighted univariate linear regression model.

Model 2 was a weighted multivariable linear regression models following backward stepwise regression analysis.

Table S6. The association between confounding variables and AFT (n = 1,406)

| Variables | Model 1 | *P* | Model 2 | *P* |
| --- | --- | --- | --- | --- |
|  | β (95% CI) |  | β (95% CI) |  |
| Age | -0.25 (-0.29, -0.20) | <0.001 | -0.22 (-0.27, -0.18) | <0.001 |
| Gender |  |  |  |  |
| Male | Ref |  |  |  |
| Female | -0.50 (-1.31, 0.31) | 0.217 |  |  |
| Race |  |  |  |  |
| Non-Hispanic White | Ref |  | Ref |  |
| Non-Hispanic Black | -3.79 (-4.80, -2.79) | <0.001 | -3.50 (-4.45, -2.55) | <0.001 |
| Mexican American | -2.79 (-3.98, -1.60) | <0.001 | -2.14 (-3.22, -1.07) | <0.001 |
| Other race | -2.91 (-4.50, -1.33) | 0.001 | -2.93 (-4.18, -1.68) | <0.001 |
| PIR |  |  |  |  |
| <1 | Ref |  |  |  |
| ≥1 | 2.98 (2.01, 3.96) | <0.001 |  |  |
| Marriage |  |  |  |  |
| Married or living with partner | Ref |  |  |  |
| Living alone | -1.25 (-2.13, -0.37) | 0.007 |  |  |
| Education |  |  |  |  |
| High school and below | Ref |  | Ref |  |
| Above high school | 3.77 (3.08, 4.45) | <0.001 | 2.45 (1.74, 3.15) | <0.001 |
| PA |  |  |  |  |
| <450 | Ref |  | Ref |  |
| 450-750 | 0.88 (-0.60, 2.36) | 0.234 | 1.15 (-0.15, 2.44) | 0.080 |
| ≥750 | 2.56 (1.28, 3.85) | <0.001 | 1.44 (0.45, 2.42) | 0.006 |
| Unknown | -0.24 (-1.44, 0.95) | 0.682 | -0.32 (-1.27, 0.64) | 0.497 |
| Smoking |  |  |  |  |
| Never | Ref |  |  |  |
| Former | -0.08 (-1.06, 0.89) | 0.865 |  |  |
| Current | -1.18 (-2.35, -0.02) | 0.047 |  |  |
| Hypertension |  |  |  |  |
| No | Ref |  |  |  |
| Yes | -1.13 (-2.10, -0.16) | 0.024 |  |  |
| Antidiabetic drug |  |  |  |  |
| No | Ref |  |  |  |
| Yes | -2.24 (-3.27, -1.21) | <0.001 |  |  |
| Dyslipidemia |  |  |  |  |
| No | Ref |  |  |  |
| Yes | -0.50 (-1.63, 0.62) | 0.368 |  |  |
| Heavy drinking |  |  |  |  |
| No | Ref |  |  |  |
| Yes | 0.97 (-0.51, 2.45) | 0.192 |  |  |
| Unknown | -1.52 (-2.40, -0.65) | 0.001 |  |  |
| BMI |  |  |  |  |
| <25 | Ref |  |  |  |
| ≥25 | 0.10 (-0.95, 1.14) | 0.851 |  |  |
| Depression |  |  |  |  |
| No | Ref |  | Ref |  |
| Yes | -1.98 (-3.07, -0.88) | 0.001 | -1.12 (-2.17, -0.08) | 0.037 |
| CKD |  |  |  |  |
| No | Ref |  |  |  |
| Yes | -2.02 (-3.11, -0.92) | 0.001 |  |  |
| Stroke |  |  |  |  |
| No | Ref |  |  |  |
| Yes | -1.01 (-2.83, 0.82) | 0.269 |  |  |
| Antiparkinson agents |  |  |  |  |
| No | Ref |  | Ref |  |
| Yes | -2.25 (-3.52, -0.97) | 0.001 | -1.84 (-3.46, -0.22) | 0.028 |
| Anxiolytics sedatives hypnotics |  |  |  |  |
| No | Ref |  |  |  |
| Yes | -0.62 (-2.64, 1.41) | 0.540 |  |  |
| Psychotherapeutic agents |  |  |  |  |
| No | Ref |  |  |  |
| Yes | -0.60 (-1.88, 0.68) | 0.348 |  |  |
| Total energy | 0.00 (0.00, 0.00) | <0.001 |  |  |
| Carbohydrate | 0.01 (0.00, 0.01) | 0.001 |  |  |
| Protein | 0.02 (0.01, 0.04) | <0.001 |  |  |
| Total fat | 0.02 (0.01, 0.03) | <0.001 |  |  |
| Total sugars | 0.01 (0.00, 0.01) | 0.012 |  |  |

AFT, Animal Fluency Test; OR, odds ratio; CI, confidence intervals, Ref., reference; PIR, poverty income ratio; PA, physical activity; BMI, body mass index; CKD, chronic kidney disease.

Model 1 was a weighted univariate linear regression model.

Model 2 was a weighted multivariable linear regression models following backward stepwise regression analysis.

Table S7. The association between confounding variables and DSST (n = 1,406)

| Variables | Model 1 | *P* | Model 2 | *P* |
| --- | --- | --- | --- | --- |
|  | β (95% CI) |  | β (95% CI) |  |
| Age | -1.09 (-1.23, -0.94) | <0.001 | -0.97 (-1.11, -0.84) | <0.001 |
| Gender |  |  |  |  |
| Male | Ref |  | Ref |  |
| Female | 4.40 (2.60, 6.19) | <0.001 | 7.11 (5.21, 9.02) | <0.001 |
| Race |  |  |  |  |
| Non-Hispanic White | Ref |  | Ref |  |
| Non-Hispanic Black | -14.34 (-17.67, -11.02) | <0.001 | -12.86 (-15.77, -9.96) | <0.001 |
| Mexican American | -15.80 (-19.19, -12.42) | <0.001 | -13.16 (-16.88, -9.45) | <0.001 |
| Other race | -9.93 (-14.16, -5.70) | <0.001 | -8.73 (-11.52, -5.94) | <0.001 |
| PIR |  |  |  |  |
| <1 | Ref |  | Ref |  |
| ≥1 | 14.87 (10.99, 18.75) | <0.001 | 7.25 (4.21, 10.28) | <0.001 |
| Marriage |  |  |  |  |
| Married or living with partner | Ref |  |  |  |
| Living alone | -4.69 (-7.09, -2.28) | <0.001 |  |  |
| Education |  |  |  |  |
| High school and below | Ref |  | Ref |  |
| Above high school | 14.43 (11.96, 16.90) | <0.001 | 8.69 (7.01, 10.37) | <0.001 |
| PA |  |  |  |  |
| <450 | Ref |  |  |  |
| 450-750 | 0.64 (-4.07, 5.36) | 0.783 |  |  |
| ≥750 | 6.11 (2.42, 9.81) | 0.002 |  |  |
| Unknown | -1.62 (-5.18, 1.94) | 0.360 |  |  |
| Smoking |  |  |  |  |
| Never | Ref |  | Ref |  |
| Former | -1.81 (-4.75, 1.13) | 0.218 | -0.77 (-3.16, 1.62) | 0.510 |
| Current | -6.49 (-10.09, -2.89) | 0.001 | -3.57 (-6.64, -0.49) | 0.025 |
| Hypertension |  |  |  |  |
| No | Ref |  |  |  |
| Yes | -7.88 (-10.90, -4.86) | <0.001 |  |  |
| Antidiabetic drug |  |  |  |  |
| No | Ref |  |  |  |
| Yes | -8.84 (-12.52, -5.16) | <0.001 |  |  |
| Dyslipidemia |  |  |  |  |
| No | Ref |  |  |  |
| Yes | -1.97 (-5.73, 1.78) | 0.292 |  |  |
| Heavy drinking |  |  |  |  |
| No | Ref |  |  |  |
| Yes | 3.55 (0.63, 6.47) | 0.019 |  |  |
| Unknown | -6.43 (-8.87, -4.00) | <0.001 |  |  |
| BMI |  |  |  |  |
| <25 | Ref |  |  |  |
| ≥25 | 0.91 (-2.04, 3.87) | 0.533 |  |  |
| Depression |  |  |  |  |
| No | Ref |  |  |  |
| Yes | -6.89 (-11.10, -2.69) | 0.002 |  |  |
| CKD |  |  |  |  |
| No | Ref |  | Ref |  |
| Yes | -9.98 (-13.39, -6.56) | <0.001 | -3.26 (-5.44, -1.08) | 0.005 |
| Stroke |  |  |  |  |
| No | Ref |  |  |  |
| Yes | -7.16 (-12.10, -2.23) | 0.006 |  |  |
| Antiparkinson agents |  |  |  |  |
| No | Ref |  |  |  |
| Yes | -5.11 (-14.45, 4.24) | 0.273 |  |  |
| Anxiolytics sedatives hypnotics |  |  |  |  |
| No | Ref |  |  |  |
| Yes | -3.10 (-9.13, 2.93) | 0.303 |  |  |
| Psychotherapeutic agents |  |  |  |  |
| No | Ref |  | Ref |  |
| Yes | -4.89 (-7.38, -2.40) | <0.001 | -5.94 (-8.05, -3.82) | <0.001 |
| Total energy | 0.00 (0.00, 0.00) | <0.001 | 0.00 (0.00, 0.00) | 0.021 |
| Carbohydrate | 0.01 (0.00, 0.02) | 0.049 |  |  |
| Protein | 0.07 (0.04, 0.10) | <0.001 |  |  |
| Total fat | 0.06 (0.04, 0.08) | <0.001 |  |  |
| Total sugars | 0.01 (-0.01, 0.04) | 0.346 |  |  |

DSST, Digit Symbol Substitution Test; OR, odds ratio; CI, confidence intervals, Ref., reference; PIR, poverty income ratio; PA, physical activity; BMI, body mass index; CKD, chronic kidney disease.

Model 1 was a weighted univariate linear regression model.

Model 2 was a weighted multivariable linear regression models following backward stepwise regression analysis.

Table S8. The multicollinearity assessment for covariates

| Variables | VIF | Df | VIF^(1/(2*Df)) |
| --- | --- | --- | --- |
| Z-score |  |  |  |
| Age | 2.0116 | 1 | 1.4183 |
| Gender | 3.5314 | 1 | 1.8792 |
| Race | 5.8501 | 3 | 1.3423 |
| PIR | 3.0207 | 1 | 1.738 |
| Education | 2.4152 | 1 | 1.5541 |
| Smoking | 4.0515 | 2 | 1.4187 |
| CKD | 3.7276 | 1 | 1.9307 |
| Psychotherapeutic agents | 2.6995 | 1 | 1.643 |
| Total energy | 2.6382 | 1 | 1.6242 |
| FBG | 4.1044 | 1 | 2.0259 |
| HbA1c | 5.5945 | 1 | 2.3653 |
| CERAD |  |  |  |
| Age | 1.6332 | 1 | 1.278 |
| Gender | 1.8145 | 1 | 1.347 |
| Race | 3.4212 | 3 | 1.2275 |
| Education | 2.7782 | 1 | 1.6668 |
| Hypertension | 1.6267 | 1 | 1.2754 |
| CKD | 1.4785 | 1 | 1.2159 |
| Anxiolytics sedatives hypnotics | 1.9121 | 1 | 1.3828 |
| FBG | 3.4169 | 1 | 1.8485 |
| HbA1c | 2.7349 | 1 | 1.6537 |
| AFT |  |  |  |
| Age | 2.0516 | 1 | 1.4323 |
| Race | 10.2186 | 3 | 1.4731 |
| Education | 1.7379 | 1 | 1.3183 |
| PA | 6.8488 | 3 | 1.3781 |
| Depression | 1.8753 | 1 | 1.3694 |
| Antiparkinson agents | 2.1742 | 1 | 1.4745 |
| FBG | 3.4938 | 1 | 1.8692 |
| HbA1c | 5.0823 | 1 | 2.2544 |
| DSST |  |  |  |
| Age | 1.4378 | 1 | 1.1991 |
| Gender | 2.7351 | 1 | 1.6538 |
| Race | 9.09 | 3 | 1.4446 |
| PIR | 5.997 | 1 | 2.4489 |
| Education | 1.8614 | 1 | 1.3643 |
| Smoking | 5.7286 | 2 | 1.5471 |
| CKD | 1.9973 | 1 | 1.4133 |
| Psychotherapeutic agents | 2.0974 | 1 | 1.4482 |
| Total energy | 4.267 | 1 | 2.0657 |
| FBG | 2.6493 | 1 | 1.6277 |
| HbA1c | 3.2874 | 1 | 1.8131 |

CKD, chronic kidney disease; PIR, poverty income ratio; FBG, fasting plasma glucose; HbA1c, glycated hemoglobin; CERAD, Consortium to Establish a Registry for Alzheimer's disease; AFT, Animal Fluency Test; PA, physical activity; DSST, Digit Symbol Substitution Test.
